# Exploring ethnic differences in the distribution of blood test results in healthy adult populations to inform earlier cancer detection: a systematic review

**DOI:** 10.1093/fampra/cmae021

**Published:** 2024-05-06

**Authors:** Ge Chen, Melissa Barlow, Liz Down, Luke Timothy Allan Mounce, Samuel William David Merriel, Jessica Watson, Tanimola Martins, Sarah Elizabeth Rose Bailey

**Affiliations:** Department of Health and Community Sciences, University of Exeter, Exeter, UK; Bristol Dental School, University of Bristol, Bristol, United Kingdom; Department of Health and Community Sciences, University of Exeter, Exeter, UK; Department of Health and Community Sciences, University of Exeter, Exeter, UK; Department of Health and Community Sciences, University of Exeter, Exeter, UK; Department of Health and Community Sciences, University of Exeter, Exeter, UK; Centre for Primary Care & Health Services Research, University of Manchester, Manchester, United Kingdom; Centre for Academic Primary Care (CAPC), Population Health Sciences, Bristol Medical School, University of Bristol, Bristol, United Kingdom; Department of Health and Community Sciences, University of Exeter, Exeter, UK; Department of Health and Community Sciences, University of Exeter, Exeter, UK

**Keywords:** Blood test, Cancer, Ethnicity, Inequality, Primary care

## Abstract

**Background:**

In primary care, health professionals use blood tests to investigate nonspecific presentations to inform referral decisions. Reference ranges for the commonly used blood tests in western countries were developed in predominately White populations, and so may perform differently when applied to non-White populations. Knowledge of ethnic variation in blood test results in healthy/general populations could help address ethnic inequalities in cancer referral for diagnosis and outcomes.

**Objective:**

This systematic review explored evidence of ethnic differences in the distribution of selected blood test results among healthy/general populations to inform future research aimed at addressing inequalities in cancer diagnosis.

**Methods:**

We searched PubMed and EMBASE to identify studies reporting measures of haemoglobin, MCV, calcium, albumin, platelet count, and CRP in nondiseased adults from at least 2 different ethnic groups. Two reviewers independently screened studies, completed data extraction and quality assessment using an adapted Newcastle-Ottawa scale. Participants were stratified into White, Black, Asian, Mixed, and Other groups. Data were synthesised narratively and meta-analyses were conducted where possible.

**Results:**

A total of 47 papers were included. Black men and women have lower average values of haemoglobin, MCV, and albumin, and higher average values of CRP relative to their White counterparts. Additionally, Black men have lower average haemoglobin than Asian men, whereas Asian women have lower average CRP values when compared with White women.

**Conclusions:**

There is evidence of ethnic differences in average values of haemoglobin, MCV, CRP, and albumin in healthy/general populations. Further research is needed to explore the reasons for these differences. Systematic review registration: CRD42021274580

**Key messages**

- Blood tests can inform assessment of undifferentiated illness in primary care.
- Differences in test values between ethnic groups could affect accurate diagnosis.
- This review found ethnic variation in Hb, MCV, CRP, and albumin in healthy adults.
- The implications of ethnic variation on diagnosis warrant further investigation.

## Introduction

Commonly used blood tests in primary care, including haemoglobin, mean cell volume (MCV), platelet count, serum calcium, liver function tests (such as albumin), and inflammatory markers (such as C reactive protein (CRP)) are known to have positive predictive values (PPV) > 1% for cancer. They are not specific for cancer diagnosis, but are used by healthcare professionals to investigate suspected cancer symptoms and nonspecific presentations to inform specialist referral decision^1,2^. For example, the National Institute for Health and Care Excellence (NICE) guidelines in England recommends specialist investigation for possible oesophageal or stomach cancer in patient with low haemoglobin level or raised platelet count^1^.

Cancer is a leading cause of death and a barrier to life expectancy worldwide^3^. Early-stage diagnosis of cancer increases the likelihood of survival compared to being diagnosed at advanced stage^4^. Timely referral for further investigation is an essential part of cancer diagnostic pathways to help achieve early diagnosis of cancer^5^. The incidence and outcomes of many cancer types vary by ethnicity in ethnically diverse countries like the United Kingdom and United States. Non-Hispanic Black Americans have the highest incidence and mortality from nearly all cancer types compared with other racial groups in the United States^6^. In the United Kingdom, Black and/or Asian groups have a higher incidence of gastrointestinal cancers, myeloma, Hodgkin lymphoma, thyroid, and prostate cancer compared to their White counterparts^7^. Furthermore, although the White population in the United Kingdom generally have higher incidence and mortality for most cancers^7,8^, the Caribbean, African, and Asian population were reported to have more advanced stage diagnosis of some cancers, namely breast, ovarian, colon, and nonsmall cell lung cancer^9^.

There is some evidence that the average values of commonly used blood tests in primary care could vary by ethnicity^10–13^. In addition, the reference ranges for blood tests used in western countries were developed in predominately White populations^10^, and so may perform differently when applied to non-White populations. Knowledge of possible variations in blood test results in healthy/general populations could help illuminate the extent of such variation and inform interventions to reduce inequalities in diagnosis of cancer and many diseases.

Currently, no study has systematically assessed the differences across ethnic groups in commonly used blood tests to assess patients presenting with possible cancer-related symptoms. Thus, this study critically examined the literature regarding ethnic differences in the distribution of nonspecific blood test results (haemoglobin, MCV, platelet count, calcium, albumin, and CRP) in healthy/general populations, with the aim of informing future research or intervention aimed at addressing inequalities in cancer diagnosis.

## Methods

### Protocol

The review was conducted in accordance with a protocol registered with the International Prospective Register of Systematic Reviews (PROSPERO) database (registration no. CRD42021274580) and followed the Preferred Reporting Items for Systematic Reviews and Meta-Analyses (PRISMA) 2020 reporting guidelines (Supplementary Appendix 1)^14^. The original protocol also included PSA and CA-125 tests. The result of the synthesis of PSA studies has been published elsewhere^13^, and the result of CA-125 could not be synthesised due to insufficient data.

### Eligibility criteria

Eligible studies reported measures of haemoglobin, MCV, serum calcium, serum albumin, platelet count, CRP, and CA-125 levels of adults from a general population without a disease diagnosis or symptoms suggestive of disease for at least 2 different ethnic groups. Studies that included the selected blood test values for only one broad ethnic group (i.e. White, Black, Asian, Other, and Mixed group^15^) were excluded. This is due to studies measuring test results in participants from different ethnicities within the same setting where potential ethnic inequalities existed/have been reported might reflect the relative differences and reduce selection, design, measurement, and reporting bias. Full inclusion and exclusion criteria in terms of population, exposure/comparator, outcome, study design, and publication type are presented in Table 1.

**Table 1.** Inclusion and exclusion criteria for study reported distributions of blood test results in ethnicities of healthy/general population.

	Inclusion	Exclusion
Population	Adults aged ≥18 years	Children/animals
	Recruited from a general population (populations selected due to age are acceptable provided this is consistent for each ethnic group)	Recruited due to disease or symptom status (e.g. cancer), medication usage, or other “nongeneral” population (e.g. healthcare workers, pregnant women, marathon runners etc.)
Exposure/comparator	More than one ethnic group reported.	Only one ethnic group reported
Outcomes	Blood test(s)* was/were stratified by broad ethnic group.	No stratification of blood test(s)* values by broad ethnic group
	Available blood test(s)* result as raw values, a pooled average (e.g. mean, median, range, centiles), or proportion above or below a certain figure	No available blood test(s)* result
Study design	Observational studies without matching	Matched observational studies^#^
	Baseline blood test(s)^*^ values from randomised controlled trials	Blood test(s)* values reported after intervention in randomised controlled trials
Publication type	Peer-reviewed, full text available in English^	Abstracts, full texts not peer-reviewed or available in English^

^*^Haemoglobin, mean cell volume, serum calcium, serum albumin, platelet count, C reactive protein, and CA-125; ^#^A sample of disease cases matched to controls of nondiseased population; ^Translation services were unavailable.

### Search strategy

A search to identify relevant published papers was conducted in PubMed and Embase on 24th September 2021 and again on 6th November 2022. The literature search applied keywords, MeSH (Medical Subject Headings), Emtree, and synonyms (and their combinations). Search terms were identified for domains including selected blood tests and ethnicity or ethnic groups. The terms searched for blood tests were: haemoglobin, mean cell volume, serum calcium, serum albumin, platelet count, C reactive protein, or CA-125. For ethnicity, we included MeSH terms: African, European, Asian, American Native, and Oceanic, MeSH terms for Ethnic Groups and Minority Groups, as well as commonly used terms to describe ethnic groups. The terms White and Black were included if they appeared within 2 words of ethnic* in an attempt to reduce nonspecific search returns. The reference lists of included studies were hand-searched to identify additional eligible studies. Full search terms are presented in Supplementary Appendix 2.

### Study records

Endnote X9 was used for the automatic detection and removal of duplicates, followed by manual review by one reviewer. The title and abstract of the retrieved papers were initially screened, and then full texts articles were screened for eligibility by 2 independent reviewers (the papers were divided into 5 groups and reviewers (GC, MB, LD, TM, JW, SWDM, and SERB) in 5 pairs screened each group independently) and conflicts were resolved by discussion with a third reviewer (LTAM). Cohen’s kappa statistic was calculated to assess interrater reliability. Following full-texts screening, an Excel data extraction form was designed and piloted on a sample of studies by the reviewers (Supplementary Appendix 3). Data extraction was also completed independently by 2 reviewers in pairs (GC, MB, LD, TM, JW, SWDM, LTAM, and SERB) and cross-referenced for discrepancies. Authors were contacted where possible to obtain unreported/missing data. The following data were extracted: number and age of patients, country of study, ascertainment of ethnicity, and blood test measures (e.g. median, mean, centiles, proportion above/below, and summarised result) for each major ethnic group and subgroups.

### Quality assessment

Since this systematic review extracted the blood test results from a variety of study designs, an adapted Newcastle-Ottawa scale for cohort studies was used to assess the quality and risk of bias of the included following the approach by Barlow et al.^13^ (Supplementary Appendix 4). Two reviewers independently assessed and scored each paper based on the selection, comparability, and outcome domains of the tool. Thus, a paper could score a maximum of nine stars. Conflicts were resolved by discussion with a third reviewer. Subsequently, each study’s quality was then classified as “good,” “fair,” or “poor” based on the Newcastle-Ottawa scale thresholds.

### Data synthesis

Three methods were applied to synthesise the results: narrative summary, harvest plots, and meta-analysis. Participants were categorised into 5 broad ethnic groups (White, Black, Asian, Mixed, and Other) based on the 2021 Census of England and Wales^15^. The original ethnic groups in selected studies were recorded in the narrative synthesis to illustrate heterogeneities within the broad ethnic groups. For studies that did not indicate statistical significance by *P*-value or narrative report between 2 ethnicities, a *t*-test of summary statistics or confidence intervals (CI) were calculated from the mean and SD or standard error where possible for comparison and to infer the significance of the outcome summary. Results were collated and summarised into a narrative synthesis following previously published guidance^16^.

Harvest plots were used to synthesise all results of the comparison with the White group for simplicity. The harvest plots provide a visual summary of findings for each blood test across all papers regardless of the reporting format used (such as mean, median, odd ratio, and narrative report)^17^. The White group was used as the reference group in the harvest plots since it was the reference group for most included studies.

Meta-analyses were conducted in Stata 17.0^18^, where comparable data were available from 2 or more studies that reported the outcomes using the same format or could be calculated into the same format (mean and SD). Effect estimates, stratified by sex for the differences in the blood tests between board ethnic groups, were pooled using absolute mean differences (MD) and their corresponding 95% confidence interval (CI). Random-effects models were used considering potential heterogeneity regarding factors such as sample sizes and countries of studies^19,20^. *I* squared (*I*^2^) and tau-squared (*T*^2^) were used for indicating heterogeneity^20^. Subgroup analyses (by countries of studies and study quality) were carried out when substantial heterogeneity was presented (*I*^2^ > 50%) if sufficient data were available^19,20^. Funnel plots and Egger’s regression tests (for testing publication bias) could not be conducted due to an insufficient number of studies^21^. Sensitivity analyses were conducted to examine the robustness of conclusions from pooled results. Meta-analyses were repeated using fixed-effects models to investigate bias as a result of possible exacerbated systematic differences between large and small studies^20^.

## Results

### Database search

**Fig. 1.**
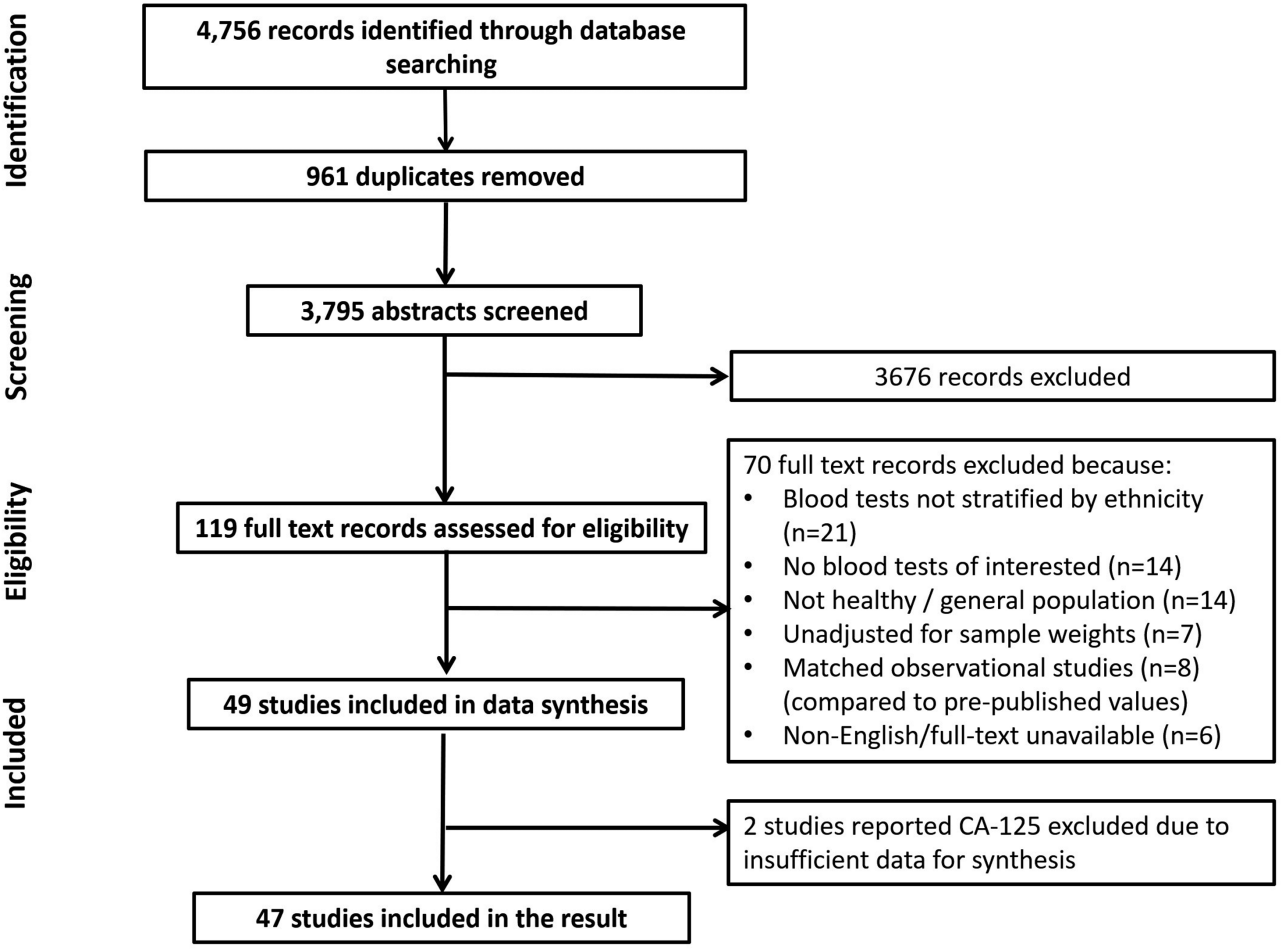
PRISMA flow diagram of the process selecting studies reported distributions of blood test results between ethnicities

Out of 4,756 studies originally retrieved from the database search, 47 were included in the data synthesis. The selection process is presented in the PRISMA flow diagram (Fig. 1). By blood test, 19 studies reported on haemoglobin, 11 on MCV, 12 on platelets, 16 on CRP, 7 on calcium, and 9 papers reported on albumin. Two eligible papers that focused on CA-125 were excluded due to insufficient data for synthesis. The level of inter-rater reliability (Cohen’s kappa) for abstract screening and full-text screening was at 0.51 (moderate) and 0.63 (substantial), respectively^22^.

### Quality assessment

Using the adapted Newcastle-Ottawa scale, 15 of the 47 studies were rated as good quality, 11 as fair, and the remaining 21 as poor (Table 2).

**Table 2.** Study quality assessment of included studies reported distributions of blood test results in ethnicities of healthy/general population using an adapted version of the Newcastle-Ottawa scale.

Study ID	Selection				Comparability	Outcome		Rating
	i	ii	iii	iv	v	vi	vii	
Akinyemiju_2019^23^	*	*	*			*		Poor
Albert_2004^24^	*	*	*		**	*	**	Good
Anand_2004^25^	*	*	*	*	**	*	**	Good
Bain_1986^26^		*			*	*	*	Poor
Bain_1996^27^		*	*		*	*		Poor
Beasley_2009^28^	*	*	*		**	*		Poor
Beutler_2005^12^	*	*	*		**	*		Poor
Bikle_1998^29^	*	*			*	*		Poor
Birk_2018^11^	*	*	*		**	*	**	Good
Brickman_1993^30^		*				*	*	Poor
Buckle_1978^31^	*	*			*	*		Poor
Chandalia_2003^32^	*	*	*	*	*	*		Poor
Cheng_2004^33^	*	*	*	*	**	*		Poor
Conroy_2011^34^	*	*	*		*	*	**	Good
Fair_2007^35^	*	*	*		**	*		Poor
Ford_2002^36^	*	*	*		**	*	**	Good
Ford_2004^37^	*	*	*		**	*	*	Good
Gader_1995^38^	*	*			*	*		Poor
Godsland_1983^39^	*	*			*	*	**	Fair
Hamson_2003^40^	*	*			*	*	*	Fair
Hanley_2007^41^	*	*	*		*	*	**	Good
Hollowell 2005^42^	*	*	*		**	*	**	Good
Horn_2002^43^	*	*	*		*	*		Poor
Jackson 1992^44^	*	*	*		*	*	**	Good
Jim_1987^45^		*			*	*		Poor
Johnson_2004^46^	*	*			*	*	**	Fair
Johnson-spear_1994^47^	*	*	*		*	*		Poor
Kerr_1982^48^	*	*	*		**	*		Poor
Khera_2005^49^	*	*	*		*	*		Poor
Kozlitina_2012^50^	*	*	*		*	*	**	Good
Lawrie_2009^51^	*	*			*	*	**	Fair
Le_2016^52^	*	*	*		**	*	*	Good
Lin_2007^53^	*	*	*			*	**	Poor
Manolio_1992^54^	*	*			*	*	**	Fair
Mast_2012^55^	*	*			*	*	**	Fair
Matthews_2005^56^		*	*		**	*	*	Fair
Miller_1988^57^		*	*		**	*	*	Fair
Nguyen_2010^58^	*	*			**	*	**	Fair
Pan_2008^59^	*	*	*		*	*	**	Good
Perry_1993^60^	*				*	*	**	Poor
Segal_2006^61^	*	*	*		**	*	**	Good
Sigola_1994^62^	*	*				*	*	Fair
Smit_2019^63^	*	*			*	*	**	Fair
Thomson_2011^64^	*	*	*		*	*	*	Good
Walter_1975^65^					*	*	*	Poor
Wener_2000^66^	*	*	*		**	*	**	Good
Yassin_2022^67^	*	*	*		*	*		Poor

i: representativeness of cohort, ii: selection of cohorts, iii: assignment of ethnicity, iv: sample size v- comparability of ethnic groups, vi: ascertainment of blood tests, and vii: statistical analysis. * 1 star, ** 2 star.

### Study characteristics

Study characteristics are presented in Supplementary Appendix 5. The majority of the studies were conducted in the United States (33/47) and the United Kingdom (6/47), with other studies conducted in Canada, Israel, Germany, South Africa, Qatar, Saudi Arabia, and Zimbabwe. Regarding ethnic groupings, the White group included non-Hispanic White, Caucasian, European, and Ashkenaz; the Black group included African American and West Indian; the Asian group included classifications of Chinese, Japanese, Filipino, Korean, Indian, Oriental, East Asian, South Asian, Gujaratis and Pacific Islander; Hispanic, Mexican American, Aboriginal, Afro-Caribbean, Bedouin, Sephardic, Native Hawaiian, Saudi, Arab, and other combined ethnics were grouped in Other; and the Mixed group included ethnicity described as Mixed by included studies.

### Narrative synthesis and meta-analyses

**Fig. 2.**
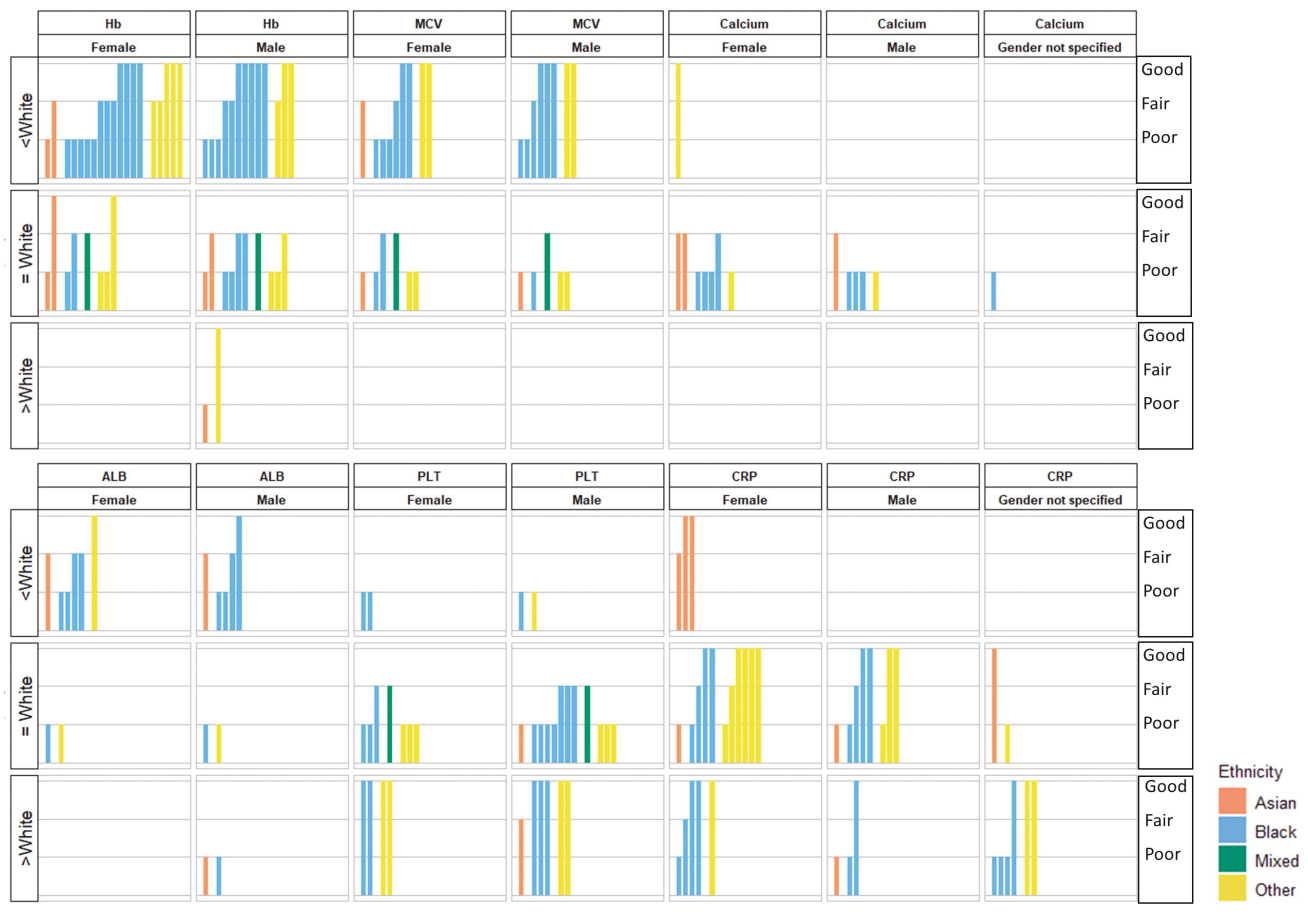
Summary of results (harvest plots) from of included studies reported comparisons between ethnic minorities and White population stratified by gender. ALB: albumin, CRP: C reactive protein; Hb: haemoglobin; MCV: mean cell volume; PLT: platelets count. In each bar graph that comprised the matrix, the hight of the bars indicated study quality of included studies (high: good, medium: fair, low: poor), and the colour of the bars indicated the minority ethnics (orange: Asian, blue: Black, green: Mixed, yellow: Other). The rows of the matrix indicated the detected differences (White) reported by each study or derived by further calculation. The columns of the matrix indicated the blood tests reported by the included studies. For example, the majority of studies (included all studies rated as good quality) reported Black men and women have lower average haemoglobin values compared with White men and women respectively.

Reported results of all included studies were summarised narratively by blood tests in Supplementary Appendix 5, which included details of datasets used, subpopulations of reported ethnicities, a summary of qualitative results (narrative report, statistical test result from each study, or estimates from secondary calculation), and quantitative results. The comparison of the blood tests between the White group and ethnic minorities by broad groups (*n* = 44) were synthesised and presented in the harvest plots illustrated in Fig. 2. Results of meta-analyses and numbers of studies included in each meta-analysis are summarised in Table 3. The findings are reported by each blood test in the following sections.

**Table 3.** Meta-analyses of studies reported distributions of blood test results between ethnicities of healthy/general population.

Blood test (unit)	Gender	Black vs White^					Asian vs White^					Black vs Asian^				
		N*	Mean difference (95% CI)	K*	T^2^	I^2^ (%)	N*	Mean difference (95% CI)	K*	T^2^	I^2^ (%)	N*	Mean difference (95% CI)	K*	T^2^	I^2^ (%)
Hb (g/dL)	Female	26549	**−0.73** **(-0.89, −0.57)**	5	**0.024**	**89.1**		n/a				1211	**−**0.08 (-0.85, 0.69)	2	0.299	95.5
	Male	30465	**−0.63** **(−0.75, −0.52)**	7	**0.013**	**77.3**	1258	0.03 (-0.34, 0.39)	2	0.054	74.9	3475	**−0.65** **(−1.10, −0.21)**	3	**0.131**	**90.1**
MCV (fL)	Female	25385	**−2.66** **(−3.68, −1.64)**	4	**0.894**	**91.4**		n/a					n/a			
	Male	24922	**−2.68** **(−3.10, − 2.27)**	4	**0.091**	**57.3**		n/a					n/a			
Platelet (x10^9^/l)	Female	6363	7.66 (−14.92, 30.25)	2	223.408	81.7		n/a					n/a			
	Male	9270	2.01 (−5.38, 9.39)	6	33.575	51.8	627	14.51 (−19.74, 48.76)	2	515.686	83.7	936	−8.8 (−17.93, 0.32)	3	53.525	40.3
CRP (mg/L)	Female	989	**0.86** **(0.17, 1.54)**	2	**0**	**0**	447	**−1.24** **(−2.01, −0.47)**	2	**0**	**0**		n/a			
	Male	4531	**0.62** **(0.07, 1.16)**	3	**0**	**0**		n/a					n/a			
Calcium (mmol/L)	Female	222	−0.06 (−0.18, 0.06)	2	0	0		n/a					n/a			
	Male	256	0.02 (−0.15, 0.20)	2	0	44.6		n/a					n/a			
Albumin (g/L)	Female		n/a					n/a					n/a			
	Male	3715	−1.14 (−5.52, 3.25)	3	13.115	88.6		n/a					n/a			

N*: Total number of participants (due to the use of the same datasets by included studies, participants might have been duplicated in the result); K*: Number of included studies; n/a: Not applicable; ^ reference group.

Bold values indicate result did not cross the null.

#### Haemoglobin:

 When comparing Black and White populations (*n* = 16), the majority of studies (13/16; all rated good quality) reported that Black men^12,31,42,44,48,50,52,55,59^ and women^12,31,42,39,43,47,48,50,52,55,59,64^ have lower average haemoglobin values compared with White men and women, respectively (Fig. 2). The meta-analyses (Table 3) results for Black vs White females (mean difference (MD) −0.73 g/dl, 95% CI: −0.89, −0.57, *I*^2^: 89.1%, *T*^2^: 0.024)^12,31,42,47,63^ and for Black vs White males (MD −0.63 g/dl 95%CI: −0.75, −0.52, *I*^2^: 77.3%, *T*^2^: 0.013)^12,31,42,44,59,63,57^ supported the narrative review finding. These results were consistent in subgroup analysis by country of studies and study quality, although, in females, the difference was larger in studies conducted in the United States than the South African study: for Black vs White females in the United States (MD: −0.84, 95% CI: −0.94, −0.75, *I*^2^: 0%, *T*^2^: 0)^12,42,47^ and in South Africa (MD: −0.45, 95% CI: −0.60, −0.31, *I*^2^: 0%, *T*^2^: 0.005)^31,63^ (Supplementary Appendix 6). Average haemoglobin levels were lower in Black men compared with Asian men (MD: −0.65, 95% CI: −1.10, −0.21, *I*^2^: 90.1%, *T*^2^: 0.131)^31,57,67^, but this was not found in women^31,67^ (Table 3).

Comparing White with Asian, Mixed, and Other groups, there was some evidence (Fig. 2) suggesting lower average haemoglobin value in the Other group than the White group regardless of sex^42,50,52,55^. Results were not suitable to be pooled due to the varied subpopulations of the Other group (e.g. Mexican-Americans, Arabs, and Hispanics) and varied reporting formats of results. No consistent evidence was found in other pairwise comparisons between ethnicities (Supplementary Appendix 5).

#### MCV:

Similar to our findings on haemoglobin, the average values of MCV were lower in Black men^12,42,50,59,43,63^ and women^12,42,50,39,43,47^ than their White counterparts. Pooled results for Black vs White groups showed a mean difference of −2.68fL (95% CI −3.10, −2.27, *I*^2^: 57.3%%, *T*^2^:0.091) for men^12,42,59,63^ and −2.66fL (95% CI: −3.68, −1.64, *I*^2^: 91.4%, *T*^2^: 0.894) for women^12,42,47,63^, respectively (Fig. 2 and Table 3). In the subgroup analysis, the differences in United States women were slightly larger (MD: −2.98, 95% CI: −4.02, −1.94, *I*^2^: 92.9%, *T*^2^: 0.772)^12,42,47^ compared with the South African study^63^ and the result was consistent in the subgroups of study quality (Supplementary Appendix 6). There was evidence from a narrative review of good quality studies that MCV values were more likely to be lower in men and women of Other ethnic groups compared with White group (Fig. 2)^42,50^.

#### Platelets:

The findings for platelets were inconsistent overall. All studies rated as good quality (3/12) suggested higher average platelet values in Black and Other groups compared with the White group^42,59,61^. However, meta-analyses result of 6 studies for men^42,59,63,57,38,62^ and 2 studies for women^42,63^ (Table 3), plus the majority of studies rated as fair or poor in the narrative synthesis^43,63,57,26,27,33,38,62^ (Fig. 2) found little evidence of ethnic differences. Although differences were shown when comparing the Black and White groups in the sensitivity analysis applying a fixed-effects model the results were either greatly driven (weight: female 96.22%, male 71.32%) by one study with a large sample size^42^. Similarly, this difference was also found in further subgroup analyses in studies conducted in the United States^42,59^ and rated as good quality^42,59^). Nevertheless, the studies all used the data from the same large dataset (National Health and Nutrition Examination Study) (Supplementary Appendix 6)^42,59^. Furthermore, other pairwise comparisons between ethnicities by both meta-analyses (Table 3) and narrative synthesis (Fig. 2) did not detect any consistent differences.

#### CRP:

Of the 13 studies that investigated the difference of average CRP value between Black and White groups, 4 found no evidence of ethnic differences^35,41,58,66^ while the remaining 9 found higher average CRP values in Black men and women^59,23,24,28,36,37,49,53,56^ (Fig. 2). Pooled estimates from 3 studies also revealed strong evidence of higher CRP values in Black men (MD: 0.62, 95%CI: 0.07, 1.16, *I*^2^: 0%, *T*^2^: 0)^59,35,58^ and women (MD: 0.86, 95%CI:0.17, 1.54, *I*^2^: 0%, *T*^2^: 0)^35,58^ (Table 3) compared with White men and women, respectively. In contrast, results of narrative synthesis of 3 studies rated as good or fair showed lower average CRP values in Asian compared to White women^35,24,34^, and the mean difference was at −1.24 mg/L (95%CI: −2.01, −0.47, *I*^2^: 0%, *T*^2^: 0) by meta-analysis^35,34^ (Table 3). The results of Asian men were limited and inconclusive^35,32^. However, we found no evidence of a difference between the Other and White groups irrespective of sex^35,66,24,56,34^ (Supplementary Appendix 5).

#### Albumin:

When comparing the Black population to the White population, the majority of studies with better quality (fair and good) reported lower average albumin values in Black men^48,59,54,60^ and women^48,39,54,60^ (Supplementary Appendix 5 and Fig. 2). However, of the studies that reported the results of men that could be pooled, there was no strong evidence of a difference^59,60,65^ (Table 3). The results of the Asian, Other and Mixed groups were not sufficient to be synthesised (Figure 2).

#### Calcium:

Findings of narrative syntheses^39,43,60,29,30^ (Supplementary Appendix 5 and Fig. 2) and meta-analyses^60,29^ (Table 3) comparing the Black group and the White group suggested no difference of average calcium value. Other pairwise comparisons between ethnic groups could not be conducted due to insufficient data.

## Discussion

This is the first systematic review investigating ethnic differences in the distribution of commonly used blood tests in primary care. There is evidence that Black men and women have lower average values in haemoglobin and MCV, and higher average values in CRP relative to their White counterparts. Asian women have lower average CRP values when compared with White women. Furthermore, there was some evidence that Black men and women have lower average albumin values compared with White, and that Black men have lower average haemoglobin values compared with Asian men.

Our findings of ethnic differences in the values of haemoglobin^68–70^, MCV^68,70,71^, albumin^68^, and CRP^72–74^ are generally consistent with the reported intervals of studies conducted with cohorts of predominately Black or Asian populations^68–70,75^. For instance, the present study found that the difference in haemoglobin levels between Black and White individuals was −0.73 g/dl (95% CI: −0.89, −0.58, *I*^2^:89.1%, *T*^2^: 0.013) and −0.63 g/dl (95%CI: −0.73, −0.52, *I*^2^: 77.3%, *T*^2^: 0.024) for females and males, respectively. This meta-analysis was conducted on studies of mainly American populations. Studies conducted on African populations also reported lower values in upper and/or lower limit of haemoglobin reference intervals (South African female:11.7-15.3g/dl, male:13.6–17.5g/dl; Nigerian female: 12.4–13.4g/dl, male:14–14.4)^70^ compared with the reported reference intervals in United States (female:12–15 g/dl, male 14–17g/dl)^76^. Moreover, studies conducted in China and Korea both noted substantially lower CRP levels in East Asian populations than studies reported the CRP levels for White population^73,74^.

These findings should be interpreted cautiously; they are likely to be influenced by more complicated reasons beyond ethnicity. The meta-analyses generally showed substantial heterogeneity (*I*^2^ > 50%, by applying random-effects models) in the results. To investigate the potential reasons for the observed differences and heterogeneity, subgroup analyses were conducted by study region and study quality where possible to test results with sufficient data (haemoglobin, MCV, and platelet). Although the effect size of *I*^2^ was slightly reduced, it remained high in the subgroup of studies conducted in the United States as well as rated at good quality. This might be due to the nature of imprecision of *I*^2^ statistics caused by varied sample sizes^77,78^ as the values of *T*^2^ in the result of haemoglobin and MCV were relatively low (Table 3)^19,20^. In addition, differences in the subpopulations within each ethnic group, such as East Asians and South Asians, and covariates such as dietary pattern, nutrient intake, and socioeconomic status could potentially influence the study findings and contribute to the unexplained heterogeneity. Therefore, we employed a narrative synthesis (Supplementary Appendix 5) to present the subpopulations and factors of covariates considered by the included studies to help our clinical interpretation.

### Clinical implications

This study only explored ethnic differences in routine blood tests amongst healthy/general populations, and therefore could not establish the association of the differences with the risk of cancer or any other disease. Nevertheless, it is possible that these differences may over or underestimate patients’ disease risk. For example, due to the lower average haemoglobin level of healthy Black individuals compared with White individuals, the risk of cancer in anaemic Black people could be diluted. The ethnic differences could be considered by future research to investigate disease risk more thoroughly and to decide whether to inform clinical guidelines. Furthermore, for clinically unexplained mild abnormal blood test results, ethnicity could be considered as a potential reason if all possible diseases could be excluded. This might help reduce the burden of over investigation.

Of note, this research was carried out in an effort to reduce health inequality and potentially raises the question of whether a patient’s ethnicity affects the propensity of the blood test result to detect disease. However any clinical recommendation on the basis of these results without full understanding of the reasons behind them could inadvertently increase health inequality. Although included studies were conducted in the general population, there are likely to be multiple factors contributing to the observed differences, and biological variation is unlikely to be the only explanatory factor. Further research is needed to explore the reasons for these differences.

### Strengths and limitations

The strength of this study was the selection of studies for ethnic comparison and the data synthesis methods. This systematic review only included studies that compared the blood test values of ethnicities from the same country rather than with the reference intervals of other countries or published guidelines, which could control for the potential bias from different healthcare systems. In addition, the findings were synthesised by sex, which is known to influence common blood test values. Considering the varied reporting formats across studies, the harvest plot qualitatively synthesised main findings, then meta-analyses validated the findings and showed the range of differences, and the narratively synthesised table summarised more detailed information about included studies.

This study also has several limitations. First, the main findings were synthesised using a broad classification of ethnic groups^15^ and the variation between subpopulations such as East and South Asians could not be compared in this study. Moreover, the role of potential covariates could not be ruled out to explain the differences observed. For example, age was a known factor that influences blood test results, synthesising by age groups was not possible due to insufficient data. In addition, although several studies reported data considered relevant factors, the adjusted models were varied and for the purpose of the original study interest. Thus, our findings were mainly based on unadjusted or age-adjusted results stratified by sex. Lastly, this study only included studies reported in English, which may have introduced language bias by excluding some studies that compared non-White populations.

### Conclusions

We found differences in the average values of haemoglobin, MCV, CRP, and albumin in healthy/general populations across different ethnic groups. Further research is required to explore the reasons for these differences and to determine whether applying race/ethnic-specific thresholds for commonly used blood tests when evaluating the risk of diseases including cancer, and making referral decisions reduces ethnic inequalities in healthcare.

## Supplementary material

Supplementary material is available at *Family Practice* online.

cmae021_suppl_Supplementary_Appendix

## Data Availability

As a systematic review, all data used is collected from other studies, no original research data was generated.
