# Supplementary material for: Exploring ethnic differences in the distribution of blood test results in healthy adult populations to inform earlier cancer detection: a systematic review
Source: Fam Pract. 2024 May 6;41(5):638–48. doi: 10.1093/fampra/cmae021 (PMC11461158; doi:10.1093/fampra/cmae021)
Supplement: cmae021_suppl_Supplementary_Appendix [file cmae021_suppl_supplementary_appendix.pdf]

Supplementary materials

Appendix 1 Preferred Reporting Items for Systematic reviews and Meta-Analyses (PRISMA) checklist 4

Appendix 2 Search criteria - Ethnicity and blood test result distribution 7

Appendix 3 Data extraction form........ 11

Appendix 4 Adapted Newcastle- Ottawa scale 14

Appendix 5 Study characteristics and summary of results by blood tests 16

Appendix 6 Meta-analyses result and forest plots 37

Haemoglobin Black vs White female (subgroup-country) 37

Haemoglobin Black vs White female (subgroup-study quality) 38

Haemoglobin Black vs White female (Fixed-effects model and random-effects model) 39

Haemoglobin Black vs White male (subgroup-country) 40

Haemoglobin Black vs White male (subgroup-study quality) 41

Haemoglobin Black vs White male (Fixed-effects model and random-effects model) 42

Haemoglobin Asian vs White male (Fixed-effects model and random-effects model) 43

Haemoglobin Black vs Asian female (Fixed-effects model and random-effects model) 44

Haemoglobin Black vs Asian male (Fixed-effects model and random-effects model) 45

MCV Black vs White female (subgroup by country of study) 46

MCV Black vs White female (subgroup by study quality) 47

MCV Black vs White female (Fixed-effects model and random-effects model) 48

MCV Black vs White male (subgroup by country of study) 49

MCV Black vs White male (subgroup by study quality) 50

MCV Black vs White male (Fixed-effects model and random-effects model) 51

Platelet (PLT) Black vs White female (Fixed-effects model and random-effects model) 52

Platelet (PLT) Black vs White male (subgroup-country of study) 53

Platelet (PLT) Black vs White male (subgroup-study quality) 54

Platelet (PLT) Black vs White male (Fixed-effects model and random-effects model) 55

Platelet (PLT) Asian vs White male (Fixed-effects model and random-effects model) 56

Platelet (PLT) Black vs Asian male (Fixed-effects model and random-effects model) 57

CRP Black vs White female (Fixed-effects model and random-effects model) 58

CRP Black vs White male (Fixed-effects model and random-effects model) 59

CRP Asian vs White female (Fixed-effects model and random-effects model) 60

Calcium Black vs White female (Fixed-effects model and random-effects model) 61

Calcium Black vs White male (Fixed-effects model and random-effects model) 61

Albumin (ALB) Black vs White male (Fixed-effects model and random-effects model) 62

References 63

Appendix 1 Preferred Reporting Items for Systematic reviews and Meta-Analyses (PRISMA) checklist

Section and Topic	Item #	Checklist item	Location where item is reported
**TITLE**			
Title	1	Identify the report as a systematic review.	1
**ABSTRACT**			
Abstract	2	See the PRISMA 2020 for Abstracts checklist.	1-2
**INTRODUCTION**			
Rationale	3	Describe the rationale for the review in the context of existing knowledge.	3-4
Objectives	4	Provide an explicit statement of the objective(s) or question(s) the review addresses.	4
**METHODS**			
Eligibility criteria	5	Specify the inclusion and exclusion criteria for the review and how studies were grouped for the syntheses.	4, 20 (table1)
Information sources	6	Specify all databases, registers, websites, organisations, reference lists and other sources searched or consulted to identify studies. Specify the date when each source was last searched or consulted.	5
Search strategy	7	Present the full search strategies for all databases, registers and websites, including any filters and limits used.	5, appendix 2
Selection process	8	Specify the methods used to decide whether a study met the inclusion criteria of the review, including how many reviewers screened each record and each report retrieved, whether they worked independently, and if applicable, details of automation tools used in the process.	5
Data collection process	9	Specify the methods used to collect data from reports, including how many reviewers collected data from each report, whether they worked independently, any processes for obtaining or confirming data from study investigators, and if applicable, details of automation tools used in the process.	6
Data items	10a	List and define all outcomes for which data were sought. Specify whether all results that were compatible with each outcome domain in each study were sought (e.g. for all measures, time points, analyses), and if not, the methods used to decide which results to collect.	4-6
	10b	List and define all other variables for which data were sought (e.g. participant and intervention characteristics, funding sources). Describe any assumptions made about any missing or unclear information.	4-6
Study risk of bias assessment	11	Specify the methods used to assess risk of bias in the included studies, including details of the tool(s) used, how many reviewers assessed each study and whether they worked independently, and if applicable, details of automation tools used in the process.	5-6
Effect measures	12	Specify for each outcome the effect measure(s) (e.g. risk ratio, mean difference) used in the synthesis or presentation of results.	6-7
Synthesis	13a	Describe the processes used to decide which studies were eligible for each synthesis (e.g. tabulating the study intervention	6-7

Section and Topic	Item #	Checklist item	Location where item is reported
methods		characteristics and comparing against the planned groups for each synthesis (item #5)).	
	13b	Describe any methods required to prepare the data for presentation or synthesis, such as handling of missing summary statistics, or data conversions.	6-7
	13c	Describe any methods used to tabulate or visually display results of individual studies and syntheses.	6-7
	13d	Describe any methods used to synthesize results and provide a rationale for the choice(s). If meta-analysis was performed, describe the model(s), method(s) to identify the presence and extent of statistical heterogeneity, and software package(s) used.	7
	13e	Describe any methods used to explore possible causes of heterogeneity among study results (e.g. subgroup analysis, meta-regression).	7
	13f	Describe any sensitivity analyses conducted to assess robustness of the synthesized results.	7
Reporting bias assessment	14	Describe any methods used to assess risk of bias due to missing results in a synthesis (arising from reporting biases).	6-7
Certainty assessment	15	Describe any methods used to assess certainty (or confidence) in the body of evidence for an outcome.	NA
**RESULTS**			
Study selection	16a	Describe the results of the search and selection process, from the number of records identified in the search to the number of studies included in the review, ideally using a flow diagram.	7, 23 (figue1)
	16b	Cite studies that might appear to meet the inclusion criteria, but which were excluded, and explain why they were excluded.	7, 23 (figue1)
Study characteristics	17	Cite each included study and present its characteristics.	8-9 Appendix 5
Risk of bias in studies	18	Present assessments of risk of bias for each included study.	7, 21(table2)
Results of individual studies	19	For all outcomes, present, for each study: (a) summary statistics for each group (where appropriate) and (b) an effect estimate and its precision (e.g. confidence/credible interval), ideally using structured tables or plots.	8-10, 22(table3)
Results of syntheses	20a	For each synthesis, briefly summarise the characteristics and risk of bias among contributing studies.	8-14 23(figue2)
	20b	Present results of all statistical syntheses conducted. If meta-analysis was done, present for each the summary estimate and its precision (e.g. confidence/credible interval) and measures of statistical heterogeneity. If comparing groups, describe the direction of the effect.	10 Appendix 6
	20c	Present results of all investigations of possible causes of heterogeneity among study results.	8-14

Section and Topic	Item #	Checklist item	Location where item is reported
			Appendix 6
	20d	Present results of all sensitivity analyses conducted to assess the robustness of the synthesized results.	Appendix 6
Reporting biases	21	Present assessments of risk of bias due to missing results (arising from reporting biases) for each synthesis assessed.	NA
Certainty of evidence	22	Present assessments of certainty (or confidence) in the body of evidence for each outcome assessed.	NA
**DISCUSSION**			
Discussion	23a	Provide a general interpretation of the results in the context of other evidence.	10-11
	23b	Discuss any limitations of the evidence included in the review.	12
	23c	Discuss any limitations of the review processes used.	12
	23d	Discuss implications of the results for practice, policy, and future research.	11-12
**OTHER INFORMATION**			
Registration and protocol	24a	Provide registration information for the review, including register name and registration number, or state that the review was not registered.	2
	24b	Indicate where the review protocol can be accessed, or state that a protocol was not prepared.	NA
	24c	Describe and explain any amendments to information provided at registration or in the protocol.	NA
Support	25	Describe sources of financial or non-financial support for the review, and the role of the funders or sponsors in the review.	14
Competing interests	26	Declare any competing interests of review authors.	14
Availability of data, code and other materials	27	Report which of the following are publicly available and where they can be found: template data collection forms; data extracted from included studies; data used for all analyses; analytic code; any other materials used in the review.	Appendix

From: Page MJ, McKenzie JE, Bossuyt PM, Boutron I, Hoffmann TC, Mulrow CD, et al. The PRISMA 2020 statement: an updated guideline for reporting systematic reviews. BMJ 2021;372:n71. doi: 10.1136/bmj.n71

For more information, visit: http://www.prisma-statement.org/

Appendix 2 Search criteria - Ethnicity and blood test result distribution

<u>Blood tests</u>

1. exp **Prostate-Specific Antigen**/
2. psa*.ti,ab.
3. prostate specific antigen*.ti,ab.
4. 1 or 2 or 3

5. exp **Platelet Count**/
6. exp **Blood Platelets**/
7. platelet*.ti,ab.
8. thrombocyt*.ti,ab.
9. 5 or 6 or 7 or 8

10. exp **Hemoglobins**/
11. haemoglobin*.ti,ab.
12. hemoglobin*.ti,ab.
13. hb.ti,ab.
14. hgb*.ti,ab.
15. anemia*.ti,ab.
16. anaemia*.ti,ab.
17. 10 or 11 or 12 or 13 or 14 or 15 or 16

18. exp **Albumins**/
19. exp **Serum Albumin**/
20. exp **Serum Albumin, Human**/
21. albumin*.ti,ab.
22. serum albumin*.ti,ab.
23. blood albumin*.ti,ab.
24. 18 or 19 or 20 or 21 or 22 or 23

25. exp **Calcium**/
26. calcium level*.ti,ab.
27. hypocalc*.ti,ab.
28. hypercalc*.ti,ab.
29. 25 or 26 or 27 or 28

30. exp **C-Reactive Protein**/
31. crp*.ti,ab.
32. c reactive protein*.ti,ab.
33. 30 or 31 or 32

34. exp **CA-125 Antigen**/
35. ca-125*.ti,ab.
36. ca125*.ti,ab.
37. cancer antigen 125*.ti,ab.
38. 34 or 35 or 36 or 37

39. **mean corpuscular volume***.ti,ab.
40. mcv*.ti,ab.
41. 39 or 40

42. exp **Blood Cell Count**/
43. exp **Hematologic Tests**/
44. full blood count*.ti,ab.
45. complete blood count*.ti,ab.
46. fbc.ti,ab.
47. cbc.ti,ab.
48. blood test*.ti,ab.
49. hematolog* test*.ti,ab.
50. 42 or 43 or 44 or 45 or 46 or 47 or 48 or 49

51. 4 or 9 or 17 or 24 or 29 or 33 or 38 or 41 or 50

Ethnic Groups

52. exp **African Continental Ancestry Group/**
53. African*.ti,ab.
54. Negro*.ti,ab.
55. Afro*.ti,ab.
56. Black adj3 ethnic*.ti,ab.
57. Black adj3 race*.ti,ab.
58. Black adj3 racial*.ti,ab.
59. 52 or 53 or 54 or 55 or 56 or 57 or 58

60. exp **European Continental Ancestry Group/**
61. Caucas*.ti,ab.
62. Europ*.ti,ab.
63. White adj3 ethnic*.ti,ab.
64. White adj3 race*.ti,ab.
65. White adj3 racial*.ti,ab.
66. 60 or 61 or 62 or 63 or 64 or 65

67. exp **Asian Continental Ancestry Group/**
68. Asian*.ti,ab.
69. Indian*.ti,ab.
70. Pakistani*.ti,ab.
71. Bangladeshi*.ti,ab.
72. Bengalis*.ti,ab.
73. Kashmiris*.ti,ab.
74. Gujaratis*.ti,ab.
75. Tamils*.ti,ab.
76. Sri Lankan*.ti,ab.
77. Chinese*.ti,ab.
78. Japanese*.ti,ab.
79. Oriental*.ti,ab.
80. Thai*.ti,ab.
81. Phillipino*.ti,ab.
82. Filipino*.ti,ab.
83. Taiwanese*.ti,ab.

84. 67 or 68 or 69 or 70 or 71 or 72 or 73 or 74 or 75 or 76 or 77 or 78 or 79 or 80 or 81 or 82 or 83

85. exp **American Native Continental Ancestry Group/**
86. Native American*.ti,ab.
87. Native Canadian*.ti,ab.
88. Native Alaskan*.ti,ab.
89. American Native*.ti,ab.
90. Canadian Native*.ti,ab.
91. Alaskan Native*.ti,ab.
92. 85 or 86 or 87 or 88 or 89 or 90 or 91

93. exp **Oceanic Ancestry Group**
94. Aborigin*.ti,ab.
95. Indigenous p*.ti,ab.
96. Maori*.ti,ab.
97. Pacific Island*.ti,ab.
98. 93 or 94 or 95 or 96 or 97

99. Inuit*.ti,ab.
100. Eskimo*.ti,ab.
101. Aleut*.ti,ab.
102. 99 or 100 or 101

103. Arab*.ti,ab.
104. Bedouin*.ti,ab.
105. Semit*.ti,ab.
106. Jew*.ti,ab.
107. Israeli*.ti,ab.
108. 103 or 104 or 105 or 106 or 107

109. Hispanic*.ti,ab.
110. Latino*.ti,ab.
111. 109 or 110

112. 59 and 66
113. 59 and 84
114. 59 and 92
115. 59 and 98
116. 59 and 102
117. 59 and 108
118. 59 and 111
119. 84 and 92
120. 84 and 98
121. 84 and 102
122. 84 and 108
123. 84 and 111
124. 92 and 98
125. 92 and 102
126. 92 and 108
127. 92 and 111

128. 98 and 102
129. 98 and 108
130. 98 and 111
131. 102 and 108
132. 102 and 111
133. 108 and 111
134. 112 or 113 or 114 or 115 or 116 or 117 or 118 or 119 or 120 or 121 or 122 or 123 or 124 or 125 or 126 or 127 or 128 or 129 or 130 or 131 or 132 or 133

135. exp **Ethnic Groups**/
136. exp **Minority Groups**/
137. ethnic*.ti,ab.
138. race*.ti,ab.
139. racial*.ti,ab.
140. 137 or 138 or 139
141. inter*.ti,ab. and 140
142. intra*.ti,ab. and 140
143. difference*.ti,ab. and 140
144. 135 or 136 or 141 or 142 or 143

145. 134 or 144

Combine

146. 51 and 145

Reference intervals

147. exp Reference Values/
148. exp Reference Standards/
149. reference value*.ti,ab.
150. reference range*.ti,ab.
151. reference interval*.ti,ab.
152. reference standard*.ti,ab.
153. distribution*.ti,ab.
154. 147 or 148 or 149 or 150 or 151 or 152 or 153

Combine

155. 145 and 154

Note:
PSA was included in the search. Then papers reported PSA test were excluded from search result at the screening stage. The result of PSA test has been published elsewhere:

M. Barlow, L. Down, L. T. A. Mounce, S. W. D. Merriel, J. Watson, T. MartinsandS. E. R. Bailey. Ethnic differences in prostate-specific antigen levels in men without prostate cancer: a systematic review. Prostate Cancer Prostatic Dis. 2023;26(2):249-256.

Appendix 3 Data extraction form

EPIC Study

Study ID:

Systematic Review Data Extraction Form

Based on https://dplp.cochrane.org/data-extraction-forms

General

1.1.	Study *(author year)*	
1.2.	Reviewer	
1.3.	Date form completed	

Methods

		**Descriptions as stated in report/paper**	**Location in text or source**
2.1.	Database or study name		
2.2.	Date of blood tests		

2.3. Notes:

Participants

		**Description**	**Location in text or source**
3.1.	Country of study		
3.2.	Classification into EPIC's ethnic groups *(e.g. African-American = Black)*		
3.3.	Ethnic distribution (n)		
3.4.	Assignment of ethnicity *(self-reported?)*		
3.5.	Blood tests reported in study		

3.6. Notes:

Outcomes

	**Blood test 1**	**Description as stated in report/paper**	**Location in text or source** *(pg & ¶/fig/table/other)*
4.1.	Blood test		
4.2.	Blood test units		
4.3.	Summary statistic reported *(e.g. mean±SD, proportion high/low, raw values)*		
4.4.	Blood test result *(stratify by ethnic group)*	*Please include values for all reported summary statistics for each ethnic group*	
	White		
	Black		
	Asian		
	Other		
	Mixed		
4.5.	Outcome summary *(e.g. White > Black > Asian)*		
4.6.	Statistical significance		

4.7. Notes:

Outcomes

	**Blood test 2**	**Description as stated in report/paper**	**Location in text or source** *(pg & ¶/fig/table/other)*
5.1.	Blood test		
5.2.	Blood test units		
5.3.	Summary statistic reported *(e.g. mean±SD, proportion high/low, raw values)*		
5.4.	Blood test result *(stratify by ethnic group)*	*Please include values for all reported summary statistics for each ethnic group*	
	White		
	Black		
	Asian		
	Other		
	Mixed		
5.5.	Outcome summary *(e.g. White > Black > Asian)*		

5.6.	Statistical significance		

5.7.	Notes:	

	**Blood test 3**	**Description as stated in report/paper**	**Location in text or source** *(pg & ¶/fig/table/other)*
6.1.	Blood test		
6.2.	Blood test units		
6.3.	Summary statistic reported *(e.g. mean±SD, proportion high/low, raw values)*		
6.4.	Blood test result *(stratify by ethnic group)*	*Please include values for all reported summary statistics for each ethnic group*	
	White		
	Black		
	Asian		
	Other		
	Mixed		
6.5.	Outcome summary *(e.g. White > Black > Asian)*		
6.6.	Statistical significance		

6.7.	Notes:	

Copy and paste the above tables if more than three blood tests per paper

Appendix 4 Adapted Newcastle- Ottawa scale

**Risk of Bias Assessment - Newcastle-Ottawa Quality Assessment Scale for Cohort Studies (adapted for EPIC)**				
Questions to be awarded with a maximum of one star, if appropriate, unless otherwise stated.				
Selection				
1.1.	Representativeness of the exposed cohort	a) truly representative of a general / healthy population *		
		b) somewhat representative of a general / healthy population *		
		c) selected group of participants e.g. hospital workers, marathon runners etc.		
		d) no description of the derivation of the cohort		
1.2.	Selection of ethnic groups	a) drawn from the same community / database *		
		b) drawn from a different source		
		c) no description of the derivation of the ethnic groups		
1.3.	Ascertainment of ethnicity	a) Self-report *		
		b) Extracted from medical records (if evidence the ethnicity originally came from self report) *		
		c) Determined by clinician or researcher, or no evidence to suggest ethnicity came from a self report		
		c) No description		
1.4.	Sample size	a) calculated and described *		
		b) not calculated or not described		
Comparability				
2.1.	Comparability	a) study controls for sex *		
	Maximum of two stars for this domain	b) study controls for age *		

		c) study controls for any additional factor		
Outcome				
3.1.	Ascertainment of blood test	a) secure records (e.g. medical records, laboratory results) *		
		b) self-report		
		e) no statement		
3.2.	Statistical test to compare differences in blood test values across ethnic groups	a) clearly described and appropriate, and the measurement of the association is presented with the probability level (p value), CIs, or mean±SD (to calculate CIs) **		
	Maximum of two stars for this question	b) statistical test used and p-value, CIs or mean±SD provided, but absent or unclear description of the statistical test used*		
		c) the statistical test is not appropriate, not described, or incomplete		
4.	Notes:			
5.	Risk of bias scoring:			Good, fair, or poor?
	Selection		/ 4	
	Comparability		/ 2	
	Outcome		/ 3	
		<u>Thresholds for converting the Newcastle-Ottawa scales to AHRQ standards (good, fair, and poor):</u>		
		**Good quality:** 3 or 4 stars in selection domain AND 1 or 2 stars in comparability domain AND 2 or 3 stars in outcome/exposure domain		
		**Fair quality:** 2 stars in selection domain AND 1 or 2 stars in comparability domain AND 2 or 3 stars in outcome/exposure domain		
		**Poor quality:** 0 or 1 star in selection domain OR 0 stars in comparability domain OR 0 or 1 stars in outcome/exposure domain		

Appendix 5 Study characteristics and summary of results by blood tests

Study ID	Blood tests	Dataset /study name	Country of study	Age (years)	Stat	Subgroup	Ethnic distribution					Quality	Covariates
							White	Asian	Black	Other	Mixed		
**#Beutler_2005** [1]	Hb	NR	USA	Mean: Female-White 57.6, Black 49.8 Male-White 57.6, Black 51.0	Count	Female<br>Male	15624<br>15405		760<br>733			Poor	Mean difference: 0.6 g/dL in age matched and iron deficiency excluded subset
					Result: g/dL (mean (SE))	Female<br>Male	13.49 (0.01)<br>14.93 (0.01)		12.7 (0.04)<br>14.45 (0.04)				
					Outcome summary	Female<br>Male	White> Black (African-American)<br>White> Black (African-American)						
**#Buckle_1978** [2]	Hb	NR	South Africa	Range: 18-50	Count	Female<br>Male	219<br>504	93<br>611	893<br>2250			Poor	
					Result: g/dL (mean (SD))	Female<br>Male	13.32 (0.98)<br>15.16 (0.97)	12.56 (0.85)<br>15.34 (1.25)	12.87 (1.12)<br>14.39 (1.21)				
					Outcome summary	Female<br>Male	White (Caucasian) >Black >Asian;<br>Asian>White (Caucasian) >Black						
**#Cheng_2004** [3]	Hb	NHANES III	USA	Range: 18 - 75	Count	Female<br>Male	1937<br>1188		1048<br>564	1185<br>550		Poor	
					Result	Female<br>Male	No numerical result was reported						
					Outcome summary	Female<br>Male	No evidence of difference was reported between White (Non-Hispanic White); Black (Non-Hispanic black) and Other (Mexican American)						
**#Godsland_1983** [4]	Hb	NR	UK	Range: 16-45	Count	Female (non-OC user)	69	117 (Oriental)+109 (Indian)	123			Fair	OC user
						Female (OC user)	84	59 (Oriental)+79 (Indian)	142				

Study ID	Blood tests	Dataset /study name	Country of study	Age (years)	Stat	Subgroup	Ethnic distribution					Quality	Covariates
							White	Asian	Black	Other	Mixed		
					Result: g/dL (mean (range))	Female (non-OC user)	12.9 (11.3 - 15)	12.46 (8.9 - 14.5 Oriental) & 12.36 (8.7 - 14.9 Indian)	12.46 (8.9 - 14.5)				
						Female (OC user)	12.8 (11.2 - 14.9)	12.52 (10.2 - 14.2 Oriental) &12.58 (9.8 - 14.1 Indian)	12.52 (10.2 -14.2)				
					Outcome summary	Female (Total)	White > Black and Asian (Indian) and Asian (Oriental), Oriental>Indian						
**#Hollowell_2005**[5]	Hb	NHAES III	USA	Range: 20 -70 years and over	Count	Female<br>Male	3642<br>3147		2400<br>2961	2166<br>3170		Good	Age group weight adjusted
					Result: g/dL (mean (SD))	Female<br>Male	13.45 (1.1)<br>14.94 (1.1)		12.53 (1.2)<br>14.38 (1.2)	13.2 (1.2)<br>15.22 (1.1)			
					Outcome summary	Female<br>Male	White (Non-Hispanic White)>Other (Mexican American)>Black (Non-Hispanic Black)<br>Other (Mexican American)>White (Non-Hispanic White)>Black (Non-Hispanic Black)						
**#Horn_2002**[6]	Hb	NHANES III	USA	NR	Count	Female<br>Male	1279<br>1032		842<br>734	909<br>1000		Poor	
					Result: g/dL (95% reference interval)	Female<br>Male	11.5 - 15.3<br>13.4 - 16.8		10.6 - 14.5<br>12.7 - 16.7	11.2 - 15.0<br>13.6 - 17.2			
					Outcome summary	Female<br>Male	White (Non-Hispanic White)>Black (Non-Hispanic Black)<br>Other (Mexican American) > Black (Non-Hispanic Black)						
**#Jackson 1992**[7]	Hb	NHANES II	USA	Range: 18-49	Count	Male	2394		303			Good	Hb below normal (13g/dl) were removed from the sample
					Result: g/dL (mean (SD))	Male	15.3 (1.0)		14.58 (1.18)				
					Outcome summary	Male	White (European American)>Black (African American)						
**#Jim_1987**[8]	Hb	NR	USA	20-40 majority	Count	Female	43	33 (Chinese) + 72 (Filipino) + 41 (Japanese)				Poor	
						Male	70	78 (Chinese) + 81 (Filipino) + 70 (Japanese)					

Study ID	Blood tests	Dataset /study name	Country of study	Age (years)	Stat	Subgroup	Ethnic distribution White	Asian	Black	Other	Mixed	Quality	Covariates
					Result: g/dL (Median (range))	Female	13.5 (11.2 - 15.9)	13.1 (11.3 - 14.3 Chinese) & 13.1 (10.9 - 15.2 Filipino) & 13.4 (11.2 - 15.7 Japanese)					
						Male	14.9 (13.2 - 16.1)	14.8 (13.5 - 16.1 Chinese) & 15.2 (13.2 - 17.4 Filipino) & 15.7 (13.6 - 18 Japanese)					
					Outcome summary	Female<br>Male	No evidence of difference<br>No evidence of difference						
**#Johnson-spear_1994** [9]	Hb	NHANES II	USA	Range: 18-44	Count	Female	2301		388			Poor	Mean remained the same after adjusting for education, income, smoking iron status
					Result: g/dL (mean (SD))	Female	13.4 (1.0)		12.6 (1.2)				
					Outcome summary	Female	White>Black						
**#Kerr_1982** [10]	Hb	HANES I	USA	Range: 18-75	Count	18-44 yr:<br>Female<br>Male	3773<br>1838		931<br>330			Poor	
						45-75yr total	5116		1008				
					Result: g/dL (mean (SE))	18-44 yr:<br>Female<br>Male	 11.5 (0.4)<br>11.3 (0.4)		 10.9 (0.6)<br>10.9 (0.6)				
						45-75yr total	11.5 (0.2)		10.8 (0.6)				

Study ID	Blood tests	Dataset /study name	Country of study	Age (years)	Stat	Subgroup	Ethnic distribution White	Asian	Black	Other	Mixed	Quality	Covariates
					Outcome summary	18-44 yr: Female<br>Male<br><br>45-75yr total	White (Hispanic included)>Black;<br>White (Hispanic included)>Black<br><br>White (Hispanic included)>Black						
**#Kozlitina_2012**[11]	Hb	Dallas Heart Study	USA	Range: 18-85	Count	Total	1065		1633	459		Good	
					Result: (% Anaemia (<12g/dL))	Female<br>Male	7.1<br>0.8		30<br>4.5	14.1<br>0.5			
					Outcome summary	Total	Anaemia was more common in Black (African) when comparing with White (European American) and Other (Hispanic) respectively; and more common in Other (Hispanic) when comparing with White (European American)						
**#Lawrie_2009**[12]	Hb	Ours	South Africa	Average 41	Count	Female			372	218		Fair	
					Result: g/dL (2.5% - 97.5% percentile)	Female			11.6 - 16.1	11.6 -16.8			
					Outcome summary	Female	Other (Combined Asian, coloured and Caucasian)>Black						
**#Le_2016**[13]	Hb	NHANES 2003-2012	USA	Range: 30-85	Count	Total	16157		9826	12156		Good	
					Result: g/dL (mean (SD) of % Anaemia^ in age groups)	Female<br>Male	10.0 (4.3)<br>10.8 (3.9)		24.3 (6.7)<br>14.6 (10.9)	11.9(2.8)<br>5.3 (3.9)			
					Outcome summary	Female<br><br>Male	Anaemia was more common in Black (Non-Hispanic Black) and Other (Hispanic) compared with White (Non-Hispanic White)<br><br>Anaemia was more common in Black (Non-Hispanic Black) and Other (Hispanic) compared with White (Non-Hispanic White)						

Study ID	Blood tests	Dataset /study name	Country of study	Age (years)	Stat	Subgroup	Ethnic distribution					Quality	Covariates
							White	Asian	Black	Other	Mixed		
**#Mast _2012** [14]	Hb	REDS-II and NHANES	USA	Range: 18-70 and over	Count	Total:<br>REDS-II<br><br><br><br>NHANES	 31127<br><br><br><br>7270		 1803<br><br><br><br>1128	 1913 (Hispanic)+ 1913 (Other)<br><br>1282 (Hispanic)+ 584 (Other)		Fair	
					Result: g/dL (mean difference, (SE))	Female:<br>REDS-II<br>NHANES<br><br>Male:<br>REDS-II<br>NHANES	Reference		 -0.48 (0.02)<br>-0.13 (0.05)<br><br><br>-0.29 (0.03)<br>-0.89 (0.05)	Hispanic:<br>-0.15 (0.02),<br>-0.4(0.05) &<br>Other:<br>-0.1 (0.02)<br>-0.29 (0.07)<br><br>Hispanic:<br>-0.14 (0.03),<br>0.01 (0.05)&<br>Other:<br>-0.07 (0.03)<br>-0.12 (0.07)			
					Outcome summary	Female:<br>REDS-II<br>NHANES<br><br>Male:<br>REDS-II<br>NHANES	 White > Hispanic/Other > Black<br>White > Hispanic/Other > Black<br><br><br>White > Hispanic/Other > Black<br>White/Hispanic/Other > Black						
**#Miller _1988** [15]	Hb	NR	UK	Range: 45-54	Count	Male	68	75	24			Fair	
					Result: g/dL (mean (SD))	Male	15.5 (1.0)	15.3 (1.2)	14.7 (1.1)				
					Outcome summary	Male	No evidence of difference between White (European), Asian (Indian) and Black (West Indian)						
**#Pan_2008** [16]	Hb	NHANES III	USA	Range: 20--65	Count	Male	1905		1600			Good	
					Result: g/dL (mean (SE))	Male	15.3 (<0.1)		14.6 (<0.1)				
					Outcome summary	Male	White (Non- Hispanic White) >Black (Non-Hispanic Black)						

Study ID	Blood tests	Dataset /study name	Country of study	Age (years)	Stat	Subgroup	Ethnic distribution					Quality	Covariates
							White	Asian	Black	Other	Mixed		
#Smit_2019[17]	Hb	NR	South Africa	Range: 18-65	Count	Female<br>Male	286<br>141		33<br>32		142<br>77	Fair	
					Result: g/dL (mean (SD))	Female<br>Male	14.0 (1.6)<br>13.4 (1.4)		13.5 (1.3)<br>13.2 (2)		13.6 (1.3)<br>13.4 (1.1)		
					Outcome summary	Female<br>Male	No evidence of difference between White (Caucasian), Black(African) and Mixed group						
¶Thomson_2011[18]	Hb	WHI-OS	USA	Range: 50-79	Count	Female	61101	2218	5413	290 (American Indian or Alaskan Native)+ 2794 (Hispanic or Latino)+ 816 (Other)		Good	Post menopause
					Result: g/dL (% Anaemia (<12g/dL))	Female	4.5	4.2	16.3	6.9 (American Indian or Alaskan Native) & 5.6 (Hispanic or Latino) & 6.9 (Other)			
					Outcome summary	Female	White (Non-Hispanic White) & Asian (Asian/pacific islander) & Other (American Indian or Alaskan Native/Hispanic/Latino/ Other)>Black (African American)						
#Yassin_2022[19]	Hb	NR	Qatar	Range: 18-60	Count	Female<br>Male		130<br>355	95<br>160	225<br>515		Poor	
					Result: g/dL (mean (SD))	Female<br>Male		12.8 (0.96)<br>14.9 (1.3)	12.4 (0.9)<br>14.5 (1.2)	12.7 (1.0)<br>14.7 (1.3)			
					Outcome summary	Female<br>Male	No evidence of difference between Asian, Black (African) and Other (Arab)<br>Asian>Black (African)						
#Beutler_2005[1]	MCV	NR	USA	Mean: Female- White 57.6, Black 49.8 Male- White 57.6, Black 51.0	Count	Female<br>Male	15624<br>15405		760<br>733			poor	Mean difference: -2.7 fL in age matched and iron deficiency excluded subset
					Result: fL (mean (SE))	Female<br>Male	90.5 (0.04)<br>90.7 (0.04)		86.8 (0.2)<br>87.7 (0.2)				
					Outcome summary	Female<br>Male	White> Black (African-American)<br>White> Black (African-American)						
#Cheng_2004[3]	MCV	NHANES III	USA	Range: 18 - 75	Count	Female<br>Male	1937<br>1188		1048<br>564	1185<br>550		Poor	

Study ID	Blood tests	Dataset /study name	Country of study	Age (years)	Stat	Subgroup	Ethnic distribution					Quality	Covariates
							White	Asian	Black	Other	Mixed		
					Result	Female<br>Male	No numerical result was reported						
					Outcome summary	Female<br>Male	No evidence of difference was reported between White (Non-Hispanic White); Black (Non-Hispanic black) and Other (Mexican American)						
**#Godsland_1983**[4]	MCV	NR	UK	Range: 16-45	Count	Female (non-OC user)	69	117 (Oriental)+ 109 (Indian)	123			Fair	OC user
						Female (OC user)	84	59 (Oriental)+ 79 (Indian)	142				
					Result: fL (mean (range))	Female (non-OC user)	89.1 (77 -99)	88.7 (75 - 101 Oriental) & 85.3 (69 - 99 Indian)	86.3 (65 - 100)				
						Female (OC user)	89.6 (81-101)	89.3 (81 - 96 Oriental) &85.7 (69 - 97 Indian)	86.9 (73 - 101)				
					Outcome summary	Female (total)	White > Black and Asian (Indian) and Asian (Oriental), Asian (Oriental)> Black, Oriental>Indian						
**#Holloway_2005**[5]	MCV	NHAES III	USA	Range: 20 -70 years and over	Count	Female<br>Male	3642<br>3147		2400<br>2961	2166<br>3170		Good	Age group weight adjusted
					Result: g/dL (mean (SD))	Female<br>Male	90.6 (4.6)<br>91.0 (4.7)		87.1 (6.45)<br>88.3 (6.12)	88.6 (5.5)<br>90.0 (4.4)			
					Outcome summary	Female<br>Male	White (Non-Hispanic White)>Other (Mexican American)>Black (Non-Hispanic Black)<br>White (Non-Hispanic White)>Other (Mexican American)>Black (Non-Hispanic Black)						
**#Horn_2002**[6]	MCV	NHANES III	USA	NR	Count	Female<br>Male	1279<br>1032		842<br>734	909<br>1000		Poor	
					Result: mmol/L (95% reference interval)	Female<br>Male	83.1 - 97.8<br>83.5 - 98		75.1 - 98.5<br>78.1 - 98.4	80.7 - 97.0<br>82.3 - 97.1			
					Outcome summary	Female<br>Male	White (Non-Hispanic White)>Black (Non-Hispanic Black)<br>White (Non-Hispanic White)>Black (Non-Hispanic Black)						

Study ID	Blood tests	Dataset /study name	Country of study	Age (years)	Stat	Subgroup	Ethnic distribution					Quality	Covariates
							White	Asian	Black	Other	Mixed		
**#Jim_1987**[8]	MCV		USA	20-40 majority	Count	Female	43	33(Chinese)+72(Filipino)+41(Japanese)				Poor	
						Male	70	78(Chinese)+81(Filipino) + 70 (Japanese)					
					Result: u[3] (Median (range))	Female	89 (82- 96)	88.2 (80.6 - 97.8 Chinese) & 89.4 (81.4 - 97.3 Filipino) & 90.5 (81-98.9 Japanese)					
						Male	89 (81.2 - 100.7)	88 (81 - 95 Chinese) & 90 (81 - 99 Filipino) & 91 (83 - 99 Japanese)					
					Outcome summary	Female Male	No evidence of difference between White (Caucasian) and Asian (Chinese, Filipino and Japanese)						
**#Johnson-spear_1994**[20]	MCV	NHANES II	USA	Range: 18-44	Count	Female	2301		388			Poor	
					Result: fL (mean (SD))	Female Iron deficiency	85.1 (5.6)		78.6 (9.3)				
						Non- iron deficiency	90.2 (5.1)		89.1 (6.6)				
					Outcome summary	Female (Total)	White>Black						
**#Kozlitina_2012**[11]	MCV	Dallas Heart Study	USA	Range: 18-85	Count	Total	1065		1633	459		Good	
					Result: (% Microcytosis (MCV<80fL) & Macrocytosis (MCV>100fL))	Female<br>Male	3.3 & 3.7<br>0.6 & 3.3		14.3 & 1.4<br>6.2 & 1.5	7.8 & 1.1<br>1.1 & 1.6			

Study ID	Blood tests	Dataset /study name	Country of study	Age (years)	Stat	Subgroup	Ethnic distribution White	Asian	Black	Other	Mixed	Quality	Covariates
					Outcome summary	Total	Microcytosis was more common in Black (African) and Other (Hispanic) when comparing with White (European American) respectively<br><br>Macrocytosis was more common in White (European American) when comparing with Black (African) and Other (Hispanic) respectively						
**#Pan_2008**[16]	MCV	NHANES III	USA	Range: 20--65	Count	Male	1905		1600			Good	
					Result: fL (mean (SE))	Male	90.2 (0.2)		88.1 (0.2)				
					Outcome summary	Male	White (Non-Hispanic White) >Black(Non-Hispanic Black)						
**#Smit_2019**[17]	MCV	NR	South Africa	Range: 18-65	Count	Female<br>Male	286<br>141		33<br>32		142<br>77	Fair	
					Result: fL (mean (SD))	Female<br>Male	89 (6)<br>90 (6)		88 (5)<br>87 (8)		89 (6)<br>90 (4)		
					Outcome summary	Female<br>Male	No evidence of difference between White (Caucasian), Black (African) and Mixed group<br>White (Caucasian)> Black (African), Mixed > Black(African)						
**Yassin_2022**[19]	MCV	NR	Qatar	Range: 18-60	Count	Female<br>Male		130<br>355	95<br>160	225<br>515		Poor	
					Result: fL (mean (SD))	Female<br>Male		83.5 (10.2)<br>83.9 (7.6)	82.9 (13.81)<br>83.6 (5.6)	83.4 (11.0)<br>83.8 (6.9)			
					Outcome summary	Female<br><br>Male	No evidence of difference in Asian, Black (African) and Other (Arab)<br><br>No evidence of difference in Asian, Black (African) and Other (Arab)						
**#Bain_1986**[21]	Platelet count	NR	UK	Median: White 25, Black (African) 29, Black (West Indian) 26	Count	Female<br><br>Male	217<br><br>148		63(West Indian)+13 (African)<br><br>10(West Indian)+12 (African)			poor	

Study ID	Blood tests	Dataset /study name	Country of study	Age (years)	Stat	Subgroup	Ethnic distribution White	Asian	Black	Other	Mixed	Quality	Covariates
					Result: x109/l (mean (95% reference interval))	Female	289 (187 - 445)		250 (166 – 377 African) & 257 (160 - 411 West Indian)				
						Male	262 (168 - 411)		216 (128 - 365 African) & 271 (210 - 351 West Indian)				
					Outcome summary	Female<br>Male	White (Caucasian)>Black (West Indian) & Black (African);<br>White (Caucasian)> Black (African), West Indian>African						
#Bain_1996 [22]	Platelet count	NR	UK	Range: 18-55	Count	Female<br>Male	100<br>100		50<br>65	51<br>51		poor	
					Result: x109/l (geometric mean (95% range))	Female	246 (169 - 358)		207 (125 - 342)	236 (149 - 374)			
						Male	218 (143 - 322)		183 (115 - 290)	196 (122 - 313)			
					Outcome summary	Female	White (Caucasian)> Black (African), Other (Afrocaribbean)> Black (African)						
						Male	White (Caucasian)> Black (African), White (Caucasian)>Other (Afrocaribbean)						
#Cheng_2004 [3]	Platelet count	NHANES III	USA	Range: 18 - 75	Count	Female<br>Male	1937<br>1188		1048<br>564	1185<br>550		Poor	
					Result	Female<br>Male	No numerical result was reported						
					Outcome summary	Female<br>Male	No evidence of difference was reported between White (Non-Hispanic White); Black (Non-Hispanic black) and Other (Mexican American)						
#Gader_1995 [23]	Platelet count	NR	Saudi Arabia	Mean (SD) White: 34.7(8.6)	Count	Male	237	247	75	487		Poor	
				Asian: 30.4 (6.3)	Result: $x10^9$/l (mean (SD))	Male	248.7 (76.8)	247.3 (66.4)	238.5 (60.4)	256.8 (71.5)			

Study ID	Blood tests	Dataset /study name	Country of study	Age (years)	Stat	Subgroup	Ethnic distribution					Quality	Covariates
							White	Asian	Black	Other	Mixed		
				Black: 26.8 (5.5) Other: 24.9 (10.8)	Outcome summary	Male	Other (Saudi) > Black (West African); No evidence of difference was reported between Asian (Southeast Asians, Koreans and Filipinos), White (Europeans and American)						
**#Hollowell 2005** [5]	Platelet count	NHAES III	USA	Range: 20 -70 years and over	Count	Female<br>Male	3641<br>3145		2400<br>2961	2166<br>3170		Good	Age group weight adjusted
					Result: x10^9/l (mean (SD))	Female<br>Male	277.1 (70.7)<br>252.7 (67.3)		1294.5(76.0)<br>261.8 (69.7)	292.7 (72.8)<br>261.6 (64.3)			
					Outcome summary	Female	Black (Non-Hispanic Black)>White (Non-Hispanic White), Other (Mexican American)>White (Non-Hispanic White)						
						Male	Black (Non-Hispanic Black)>White (Non-Hispanic White), Other (Mexican American)>White (Non-Hispanic White)						
**#Horn_2002** [6]	Platelet count	NHANES III	USA	NR	Count	Female<br>Male	1279<br>1032		842<br>734	909<br>1000		Poor	
					Result: x10^9/l (95% reference interval)	Female<br>Male	172-398		181 -1433	175 - 431			
					Outcome summary	Female<br>Male	No evidence of difference was reported between White (Non-Hispanic White), Black (Non-Hispanic black) and Other (Mexican American)						
**#Miller_1988** [15]	Platelet count	NR	UK	Range: 45-54	Count	Male	68	75	24			Fair	
					Result: x10^3/mm^3 (mean (SD))	Male	218.3 (63)	252.0 (86.4)	217.1 (57.1)				
					Outcome summary	Male	Asian (Indian)>White (European); No evidence of difference in comparisons with Black (West Indian)						
**#Pan_2008** [16]	Platelet count	NHANES III	USA	Range: 20--65	Count	Male	1905		1600			Good	
					Result: x10^3/mm^3 (mean (SE))	Male	258.2 (2.8)		263.3 (2.4)				
					Outcome summary	Male	Black (Non-Hispanic Black)>White (Non-Hispanic White)						

Study ID	Blood tests	Dataset /study name	Country of study	Age (years)	Stat	Subgroup	Ethnic distribution					Quality	Covariates
							White	Asian	Black	Other	Mixed		
**#Segal_2006** [24]	Platelet Count	NHANES III	USA	Above 17	Count	Total	4978		3278	3400		Good	Evidence remained the same when adjusted (nutritional and inflammatory covariates and alcohol use
					Result: × 10³/μl (median (range) of geometric mean in age groups)	Female<br>Male	275 (254 - 284)<br>251 (232 - 260)		287 (265 -300)<br>262 (242 - 271)	285 (268 - 295)<br>261 (241 - 270)			
					Outcome summary	Female	Black (Non-Hispanic black) >White (Non-Hispanic White), Other (Mexican American)>White (Non-Hispanic White)						
						Male	Black (Non-Hispanic black) >White (Non-Hispanic White), Other (Mexican American)>White (Non-Hispanic White)						
**#Sigola_1994** [25]	Platelet count	NR	Zimbabwe	Range: 20-50	Count	Male	29		56			Fair	
					Result: x10⁹/l (mean (SE))	Male	263 (11)		258 (8)				
					Outcome summary	Male	No evidence of difference was reported						
**#Smit_2019** [17]	Platelet count	NR	South Africa	Range: 18-65	Count	Female<br>Male	286<br>141		33<br>32		142<br>77	Fair	
					Result: x10⁹/l (mean (SD))	Female<br>Male	267.2 (65.9)<br>284.4 (68.8)		261.2 (53.2)<br>263.6 (71.5)		270.7 (66.2)<br>273.0 (64.7)		
					Outcome summary	Female<br>Male	No evidence of difference between White (Caucasian), Black (African) and Mixed group						
**#Yassin_2022** [19]	Platelet count	NR	Qatar	Range: 18-60	Count	Female<br>Male		130<br>355	95<br>160	225<br>515		Poor	
					Result: x10⁹/l (mean (SD))	Female<br>Male		265.0 (61.8)<br>252.4 (65.7)	243.1 (43.8)<br>247.7 (63.3)	260.9 (59.5)<br>250.7 (65.2)			

Study ID	Blood tests	Dataset /study name	Country of study	Age (years)	Stat	Subgroup	Ethnic distribution White	Asian	Black	Other	Mixed	Quality	Covariates
					Outcome summary	Female<br>Male	No evidence of difference between Asian, Black (African) and Other (Arab)						
**¶Akinyemiju_2019** [26]	CRP	REGARD	USA	> 45	Count	Gender not specified (55% Female)	928		928			Poor	
					Result: % in Tertiles of sample CRP value	Gender not specified (55% Female)			Tertile 1: 32.8<br>Tertile 3: 50.9				
					Outcome summary	Gender not specified (55% Female)	Black (Non-Hispanic black) >White (Non-Hispanic white)						
**~Albert_2004** [27]	CRP	Women's Health Study	USA	Mean (SD): 54 (7.1)	Count	Female	24455	357	475	254		Good	Evidence remained the same when applying fully adjusted model*
					Result: mg/L (Median (IQR))	Female	2.01 (0.81 – 4.37)	1.12 (0.48-2.25)	2.96 (1.19 - 5.86)	2.06 (0.88 - 4.88)			
					Outcome summary	Female	Black> White> Asian (Asian/Pacific Islander)						
**#Anand_2004** [28]	CRP	SHARE	Canada	Mean (SD): 50.4 (10.3)	Count	Gender not specified (51% Female)	332	306 (Chinese)+ 323 (South Asian)		299		Good	Adjusted for age and sex
					Result: mg/L (mean (SE))	Gender not specified (51% Female)	2.1 (0.1)	1.2(0.1 Chinese) & 2.6 (0.1 South Asian)		3.7 (0.1)			
					Outcome summary	Gender not specified (51% Female)	Other (Aboriginal)>Asian (South Asian) and White (European) and Asian (Chinese), South Asian>White (European)& Chinese						
**#Beasley_2009** [29]	CRP	Health ABC	USA	Range:70-79	Count	Female<br>Male	757<br>831		592<br>471			poor	
					Result: pg/mL (median (IQR))	Female<br>Male	1.6 (1-3.1)<br>1.3 (0.9 - 2.3)		2.2 (1.1 - 4)<br>1.8 (1.1 - 3.3)				

Study ID	Blood tests	Dataset /study name	Country of study	Age (years)	Stat	Subgroup	Ethnic distribution White	Asian	Black	Other	Mixed	Quality	Covariates
					Outcome summary	Female<br>Male	Black>White,<br>Black >White						
**#Chandalia_2003**[30]	CRP	NA	USA	Mean (SD):<br>Asian 31 (2)<br>White 29 (7)	Count	Male	82	55				Poor	Evidence remained the same adjusted for total fat mass, WC, insulin area under curve, estimate
					Result: mg/L (Geometric mean)	Male	0.63	0.94					
					Outcome summary	Male	Asian (Asian Indian)> White (Caucasian)						
**~Conroy_2011**[31]	CRP	a nutritional intervention study	USA	Range: 35-47	Count	Female	67	74		23		Good	Premenopausal
					Result: mg/L (mean (SD)	Female	1.7 (3.3)	0.6 (1.5)		1.1 (1.5)			
					Outcome summary	Female	White (Caucasian)>Asian (Japanese/Chinese/Filipino), no evidence of difference when comparing with Other (Native Hawaiian/Mix)						
**#Fair_2007**[32]	CRP	Kaiser Permanente of Northern Caifornia	USA	Range: 60-69	Count	Female<br>Male	280<br>443	26<br>41	34<br>71	29<br>44		poor	
					Result: mg/L (mean (SD)	Female<br>Male	4.3 (7)<br>2.6 (6)	2.5 (4)<br>2.5 (8)	6.1 (8)<br>3.2 (5)	4.9 (6)<br>2.2 (2)			
					Outcome summary	Female<br>Male	No evidence of difference in comparison with White, Asian (east Asian), Black (African-American) and Other (Hispanic)						
**#Ford_2002**[33]	CRP	NHAHES III	USA	≥20	Count	Gender not specified	NR		NR	NR		Good	Fully adjusted model for

Study ID	Blood tests	Dataset /study name	Country of study	Age (years)	Stat	Subgroup	Ethnic distribution					Quality	Covariates
							White	Asian	Black	Other	Mixed		
					Result: (OR (95%CI))	Gender not specified	Reference		1.55 (1.31 - 1.83)	1.3 (1.06 - 1.59 Mexican American) & 1.04 (0.78 - 1.37 Other)			testing association for CRP and leisure-time physical activity **
					Outcome summary	Gender not specified	Black (African-American) and Other (Mexican-American)>White						
**#Ford_2004** [34]	CRP	NHANES 1999-2000	USA	≥20	Count	Female	963		419	618 (Mexican-American)+183 (Other)		Good	Adjusted for age; No evidence of difference between White and Black when applying fully adjusted model***
					Result: mg/L (Geometric mean)	Female	2.3		3.1	3.5			
					Outcome summary	Female	Black (African American)>White, Other (Mexican American)>White						
**#Hanley_2007** [35]	CRP	IRAS Family Study	USA	Mean (SD) Female: Black 40.44(12.96), Other 41.61 (13.49), Male: Black 42.37(14.25), Other 40.01(14.42)	Count	Female<br>Male			299<br>233	662<br>452		Good	
					Result: mg/L (median (IQR))	Female<br>Male			2.6 (0.9 - 6.0)	2.2 (0.9 - 5.1)			
					Outcome summary	Female<br>Male	No evidence of difference between Black(African-American) and Other (Hispanic)						
**#Khera_2005** [36]	CRP	Dallas Heart Study	USA	Range:30-65	Count	Female<br>Male	516<br>475		1018<br>740			Poor	
					Result: mg/L (median)	Female<br>Male	3.2<br>1.7		3.5<br>2.1				
					Outcome summary	Total	Black>White						

Study ID	Blood tests	Dataset /study name	Country of study	Age (years)	Stat	Subgroup	Ethnic distribution					Quality	Covariates
							White	Asian	Black	Other	Mixed		
**#Lin_2007**[37]	CRP	NHANES 1999-2002	USA	≥40	Count	Gender not specified (total)	3526		1252	1422		Poor	
					Result: (% of people with elevated CRP ≥10 mg/L	Gender not specified (without diabetes)	9.7		17.4	11.4			
					Outcome summary	Gender not specified (without diabetes)	Black >White, No evidence of difference in comparison with Other (Mexican American)						
**#Matthews_2005**[38]	CRP	SWAN	USA	Range: 42-52	Count	Female	1400	231 (Chinese) + 248 (Japanese)	729	226		Fair	No evidence of difference between White and Asian When applying fully adjusted model****
					Result: mg/L (median (IQR))	Female	1.4 (0.6- 3.9)	0.7 (0.3 - 1.4 Chinese) & 0.5 (0.2 - 1.2)	3 (1 -7.2)	2.3 (1 - 5.1)			
					Outcome summary	Female	Black (African-American)> White >Asian (Chinese and Japanese) and, Other (Hispanic) > Asian (Chinese and Japanese)						
**#Nguyen_2010**[39]	CRP	Bogalusa Heart Study	USA	Mean 36.3	Count	Female<br>Male	454<br>374		221<br>135			Fair	
					Result: mg/L (mean (SE))	Female<br>Male	3.3 (0.2)<br>2 (0.1)		4.1 (0.3)<br>2.6 (0.3)				
					Outcome summary	Female<br>Male	No evidence of difference between White and Black						
**#Pan_2008**[16]	CRP	NHANES III	USA	Range: 20--65	Count	Male	1905		1600			Good	
					Result: mg/dL (mean (SE))	Male	0.3 (<0.1)		0.4 (<0.1)				
					Outcome summary	Male	Black (Non-Hispanic Black)>White (Non- Hispanic White)						

Study ID	Blood tests	Dataset /study name	Country of study	Age (years)	Stat	Subgroup	Ethnic distribution					Quality	Covariates
							White	Asian	Black	Other	Mixed		
#Wener_2000[40]	CRP	NHANES III	USA	Range: 20-70 and above	Count	Female<br>Male	NR		NR	NR		Good	
					Result: mg/L (median (range) of CRP 95% percentile in age groups)	Female<br>Male	1.37 (1.04 - 1.68)<br>0.9 (0.51 - 1.24)		2.19 (1.6 - 2.56)<br>1.82 (0.73 - 2.4)	1.73 (1.44 - 2.47)<br>1.29 (0.66 - 2.59			
					Outcome summary	Female<br>Male	No evidence of difference in comparison with White (Non-Hispanic white); Black (Non-Hispanic black) and Other (Mexican American)						
#Birk_2018[41]	Albumin	Soroka medical Centre	Israel	Range: 18-52	Count	Male	118			108 (Ashkenazi)+ 255 (Sephardic)		Good	
					Result: g/L (mean (SD))	Male	40 (0.6)			39.4 (0.4 Sephardic) & 37.8 (0.4 Bedouin)			
					Outcome summary	Male	White (Ashkenazi)>Other (Sephardic)>Other (Bedouin)						
#Godsland_1983[4]	Albumin	NR	UK	Range: 16-45	Count	Female	71	37 (Oriental)+ 71 (Indian)	109			Good	
					Result: g/L (median (range))	Female	42.5 (36 - 52)	41.9 (38 - 46 Oriental) & 41.2 (34 - 48 Indian)	41.2 (35 - 49)				
					Outcome summary	Female	White > Black, White > Asian (Indian)						
#Horn_2002[6]	Albumin	NHANES III	USA	NR	Count	Female<br>Male	1279<br>1032		842<br>734	909<br>1000		Poor	
					Result: g/dL (95% reference interval)	Female<br>Male	35 - 47<br>37 - 50		32 - 47<br>36 - 49	34 - 48<br>38 - 50			
					Outcome summary	Female<br>Male	No evidence of difference in comparison with White (Non-Hispanic White), Black (Non-Hispanic Black) and Other (Mexican American)						
#Johnson_2004[20]	Albumin	NR	UK	Range: 21-62	Count	Male	141	43				Fair	

Study ID	Blood tests	Dataset /study name	Country of study	Age (years)	Stat	Subgroup	Ethnic distribution					Quality	Covariates
							White	Asian	Black	Other	Mixed		
					Result: g/dL (lower limit and upper limit)	Male	40.1 - 49.4	38.3 - 49.4					
					Outcome summary	Male	White (Caucasian> Asian (Indian)						
**#Kerr_1982** [10]	Albumin	HANES I	USA	Range: 18-44	Count	18-44 yr:<br>Female<br>Male<br><br>45-75yr total	 3773<br>1838<br><br>5116		 931<br>330<br><br>1008			Poor	
					Result: (% (SE) below standard (35g/L))	18-44 yr:<br>Female<br>Male<br><br>45-75yr total	 0.4 (0.1)<br>0.1 (0.1)<br><br>1.0 (0.01)		 1.1 (0.6)<br>0.1 (1.1)<br><br>2.1 (0.03)				
					Outcome summary	18-44 yr:<br>Female<br>Male<br><br>45-75yr total	 White (Hispanic included)>Black<br>White (Hispanic included)>Black<br><br>White (Hispanic included)>Black						
**#Manolio_1992** [42]	Albumin	CARDIA	USA	Range: 18-30	Count	Female<br>Male	1299<br>1161		1447<br>1143			Fair	
					Result: g/L (Median (5%-95%percentile))	Female<br>Male	47 (41 - 50)<br>48 (44 - 52)		45 (40 - 52)<br>47 (42 - 49)				
					Outcome summary	Female<br>Male	White>Black						
**#Pan_2008** [16]	Albumin	NHANES III	USA	Range: 20--65	Count	Male	1905		1600			Good	
					Result: mg/dL (mean (SE))	Male	4.4 (<0.1)		4.1 (<0.1)				
					Outcome summary	Male	White (Non- Hispanic White) >Black (Non-Hispanic Black)						

Study ID	Blood tests	Dataset /study name	Country of study	Age (years)	Stat	Subgroup	Ethnic distribution					Quality	Covariates
							White	Asian	Black	Other	Mixed		
**#Perry_1993** [43]	Albumin,	SHEP and CHIPS	USA	68-93	Count	Female<br>Male	18<br>25		24<br>8			Poor	
					Result: mg/dL (mean (SE))	Female<br>Male	4.74 (0.05)<br>4.62 (0.06)		4.13 (0.09)<br>4.16 (0.17)				
					Outcome summary	Female<br>Male	White (Caucasian) >Black (African-American)<br>White (Caucasian) >Black (African-American)						
**#Walter_1975** [44]	Albumin	NR	Germany	NR	Count	Male	100	100	100			Poor	
					Result: mg/ml (mean (SD))	Male	44.4 (4.6)	54.3 (15.1)	47.0 (6.8)				
					Outcome summary	Male	Black> White (German), Asian (Indian) >White (German), Asian (Indian)>Black						
**#Bikle_1998** [45]	Calcium	CARDIA	USA	Range: 25-36	Count	Female<br>Male	84<br>114		96<br>109			Poor	
					Result: mmol/L (mean (SD))	Female<br>Male	2.34 (0.12)<br>2.40 (0.12)		2.32 (0.11)<br>2.39 (0.11)				
					Outcome summary	Female<br>Male	No evidence of difference in comparison with White and Black						
**#Birk_2018** [41]	Calcium	Soroka medical Centre	Israel	Range: 18-52	Count	Male	109			110 (Bedouin)+ 253 (Sephardic)		Good	
					Result: mmol/L (mean (SD))	Male	2.31 (0.1)			2.29 (0.12 Sephardic) & 2.27 (0.1 Bedouin)			
					Outcome summary	Male	White (Ashkenazi)>Other (Bedouin)						
**#Brickman_1993** [46]	Calcium	NR	USA	Mean (SD)White: 38 (1.9)<br>Black: 39 (1.7)	Count	Female<br>Male	14<br>20		19<br>12			poor	
					Result: mg/dL (mean (SE))	Gender not specified	9.5 (0.06)		9.56 (0.07)				
					Outcome summary	Gender not specified	No evidence of difference Between White and Black						

Study ID	Blood tests	Dataset /study name	Country of study	Age (years)	Stat	Subgroup	Ethnic distribution					Quality	Covariates
							White	Asian	Black	Other	Mixed		
**#Godsland_1983**[4]	Calcium	NR	UK	Range: 16-45	Count	Female	70	36 (Oriental)+ 70 (Indian)	102			Good	
					Result: mmol/L (median (range))	Female	2.34 (2.03 - 2.45)	2.18 (2.03 -2.41 Oriental) & 2.22 (1.94 - 2.54 Indian)	2.24 (1.98 - 2.51)				
					Outcome summary	Female	No evidence of difference in comparison with White, Black and Asian (Oriental &Indian)						
**#Hamson_2003**[47]	Calcium	NR	UK	Range: 20-40	Count	Female<br>Male	51<br>37	71<br>42				Fair	
					Result: mmol/L (mean (SE))	Female<br>Male	2.2 (0.11)<br>2.2 (0.10)	2.2 (0.11)<br>1.1 (0.10)					
					Outcome summary	Female<br>Male	No evidence of difference in comparison with White and Asian (Gujaratis)						
**#Horn_2002**[6]	Calcium	NHANES III	USA	NR	Count	Female<br>Male	1279<br>1032		842<br>734	909<br>1000		Poor	
					Result: mmol/L (95% reference interval)	Female<br>Male	2.1 - 2.5<br>2.15 - 2.53		2.1 - 2.5<br>2.18 - 2.55	2.1 - 2.45<br>2.13 - 2.53			
					Outcome summary	Female<br>Male	No evidence of difference in comparison with White (Non-Hispanic White), Black (Non-Hispanic Black) and Other (Mexican American)						
**#Perry_1993**[43]	Calcium	SHEP and CHIPS	USA	Range: 68-93	Count	Female<br>Male	18<br>25		24<br>8			Poor	
					Result: mg/dL (mean (SE))	Female<br>Male	8.19 (0.07)<br>8.06 (0.07)		8.73 (0.09)<br>8.80 (0.14)				
					Outcome summary	Female<br>Male	Black (African-American)> White (Caucasian)<br>Black (African-American)> White (Caucasian)						

BMI: Body mass index; CRP: C reactive protein; Hb: haemoglobin; IQR: inter quartile range; MCV: mean cell volume HDL: high-density lipoproteins cholesterol; LDL-C: low-density lipoproteins cholesterol; MCV: mean cell volume; NR: Not reported; OR: odds ratio; PA: physical activity; SD: standard deviation; SE: standard error; SEM: standard error of the mean; TG: triacylglycerol; TC: total cholesterol; WC: Waist circumference; 95% CI: 95% confidence interval.

^Anaemia :<12g/dL for female, <13/dL for male;

Study designs: #cross-sectional study, ¶cohort study, ~trials

* Adjusted Age BMI, history of hypertension, smoking status, diabetic status, alcohol use, exercise, history of myocardial infraction in mother and/or father, estrogen use, education, triglycerides, HDL,LDL

*Adjusted for age, sex, ethnicity, education, working status, smoking status, serum cotinine concentration, hypertension, body mass index, waist-to-hip ratio, total cholesterol concentration, high-density lipoprotein cholesterol concentration, aspirin use;

**Adjusted for education, smoking status, total cholesterol concentration, systolic blood pressure, waist circumference, alcohol use, and hormone replacement therapy;

*** Adjusted for location, education, leisure PA, total calories and percent calories from fat intake.

Appendix 6 Meta-analyses result and forest plots

(IV: Weights are from fixed-effects model; DL: Weights are from random-effects model)

Haemoglobin Black vs White female (subgroup-country)

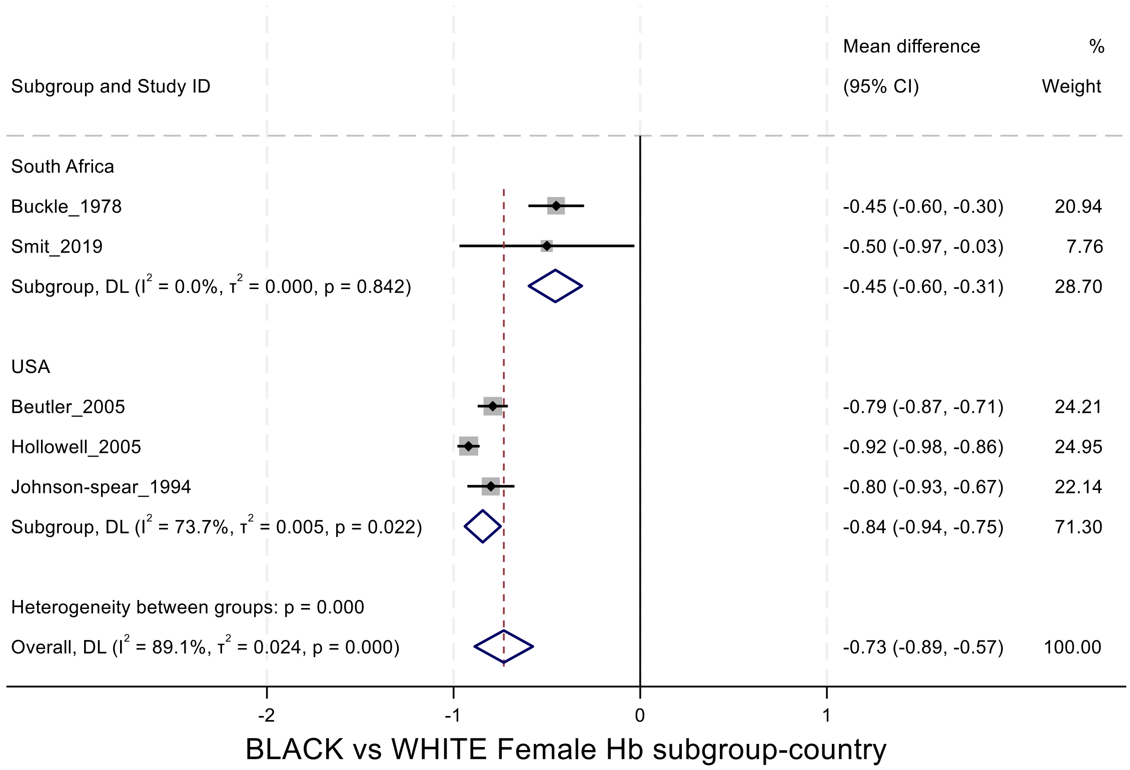

Haemoglobin Black vs White female (subgroup-study quality)

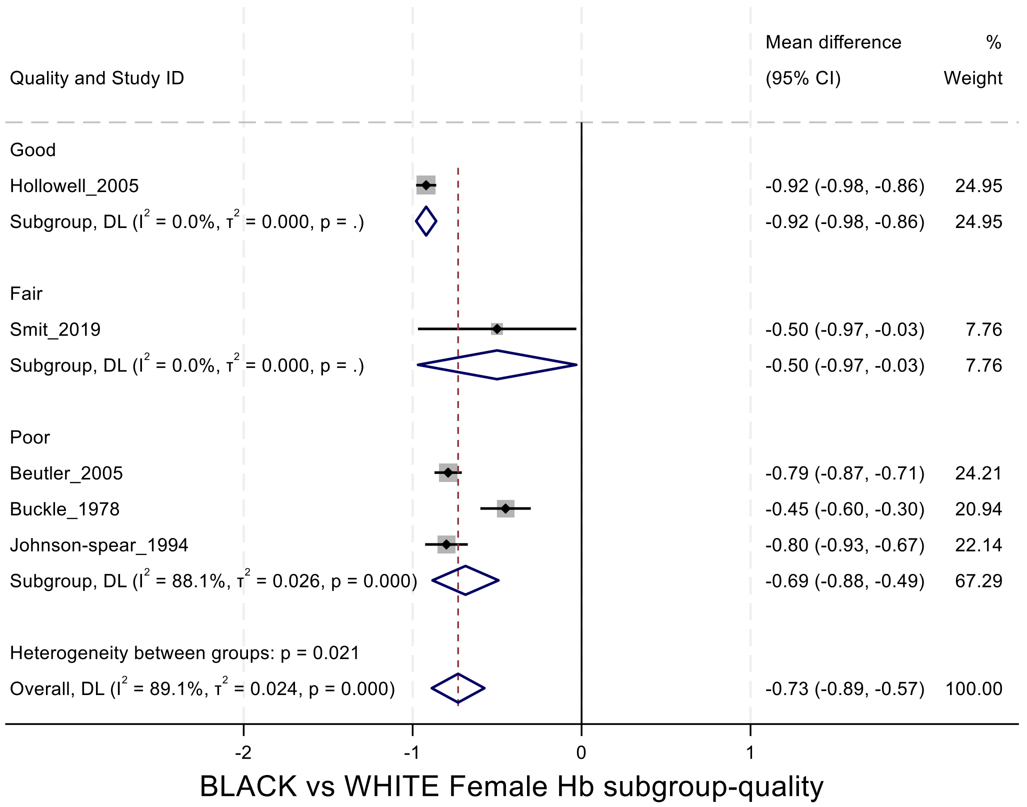

Haemoglobin Black vs White female (Fixed-effects model and random-effects model)

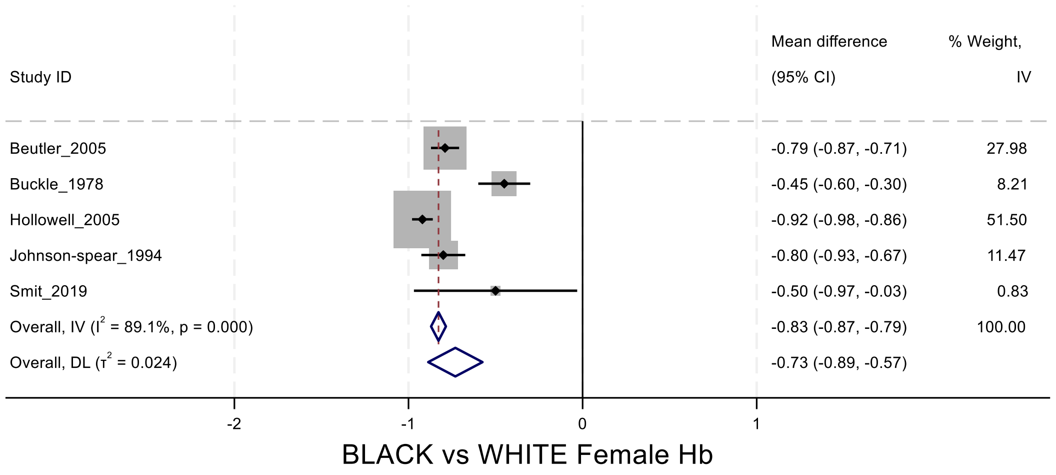

Haemoglobin Black vs White male (subgroup-country)

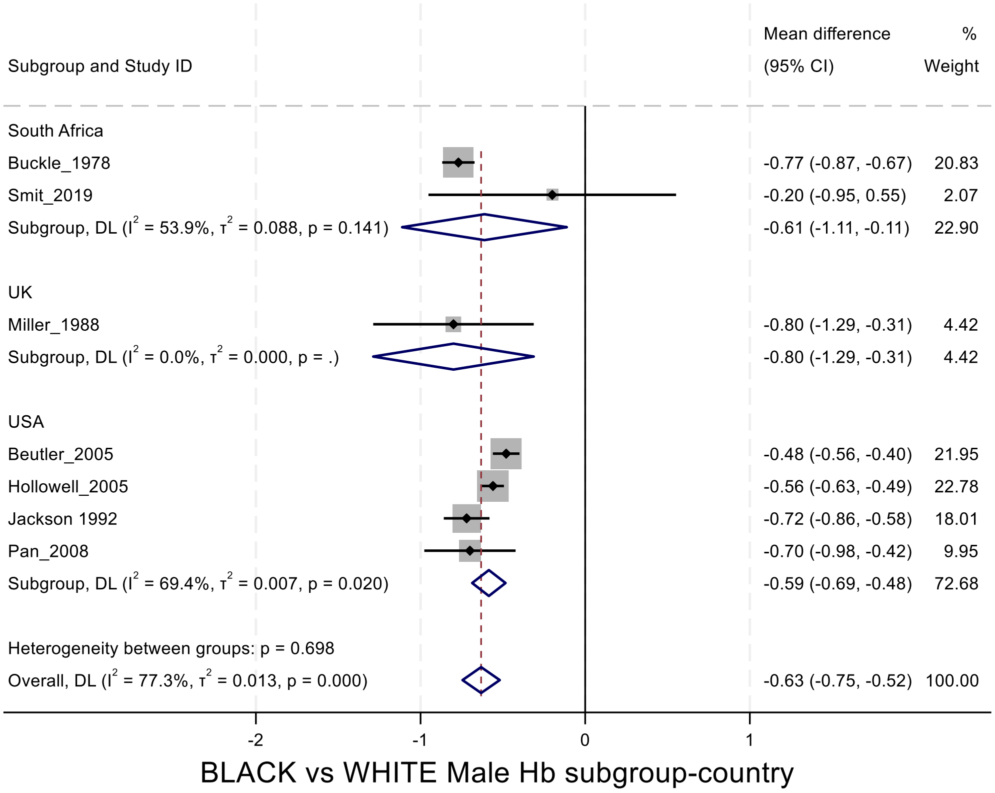

Haemoglobin Black vs White male (subgroup-study quality)

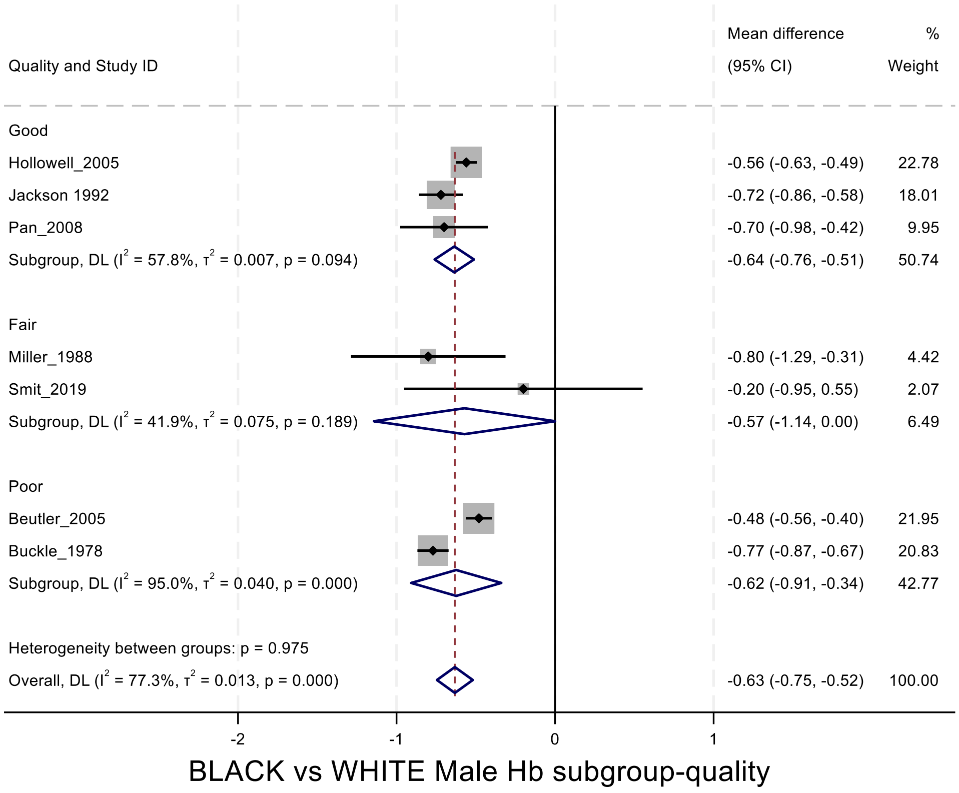

Haemoglobin Black vs White male (Fixed-effects model and random-effects model)

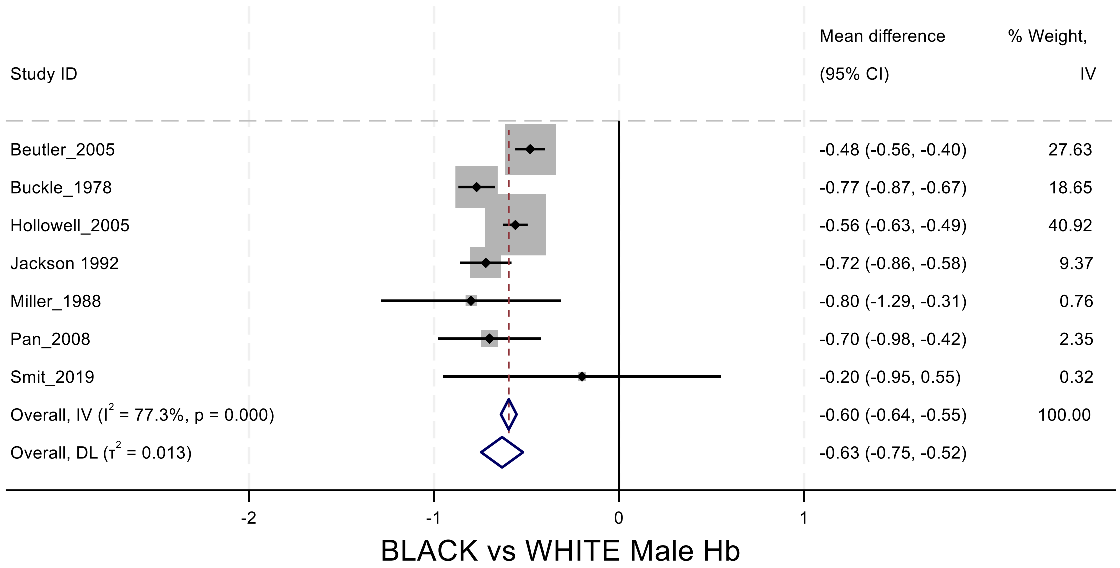

Haemoglobin Asian vs White male (Fixed-effects model and random-effects model)

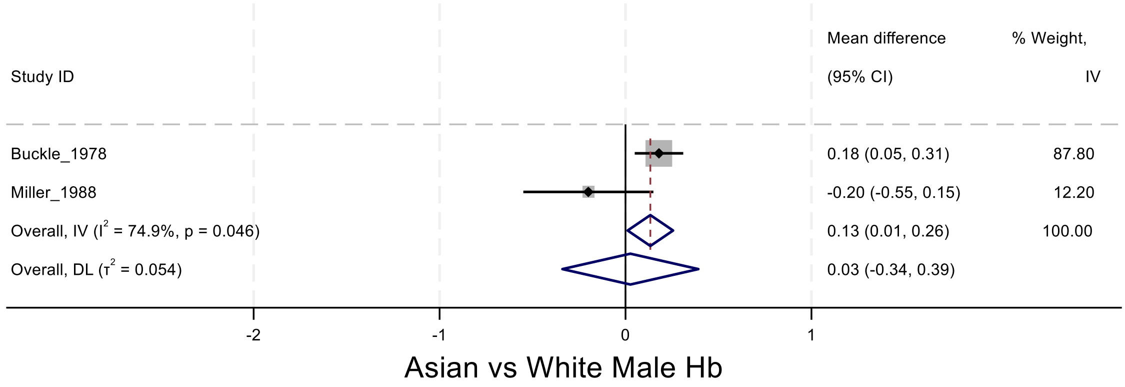

Haemoglobin Black vs Asian female (Fixed-effects model and random-effects model)

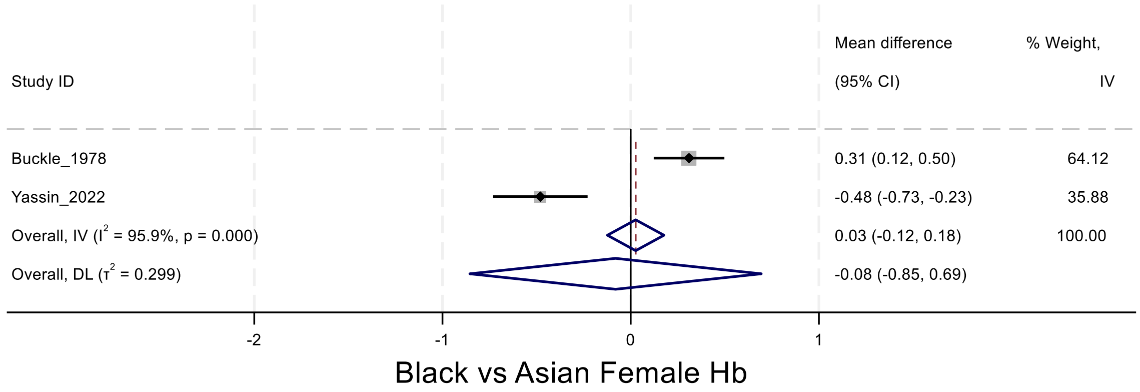

Haemoglobin Black vs Asian male (Fixed-effects model and random-effects model)

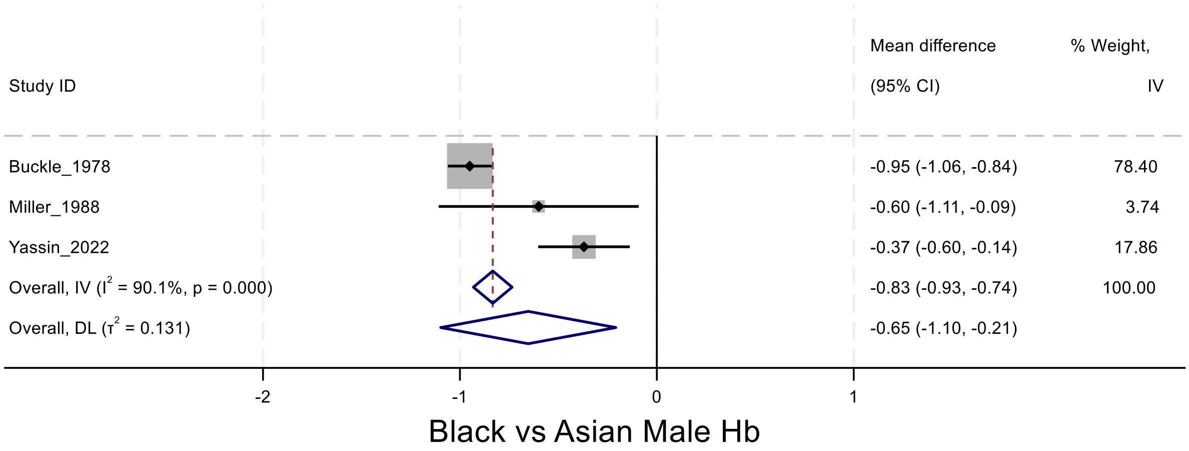

MCV Black vs White female (subgroup by country of study)

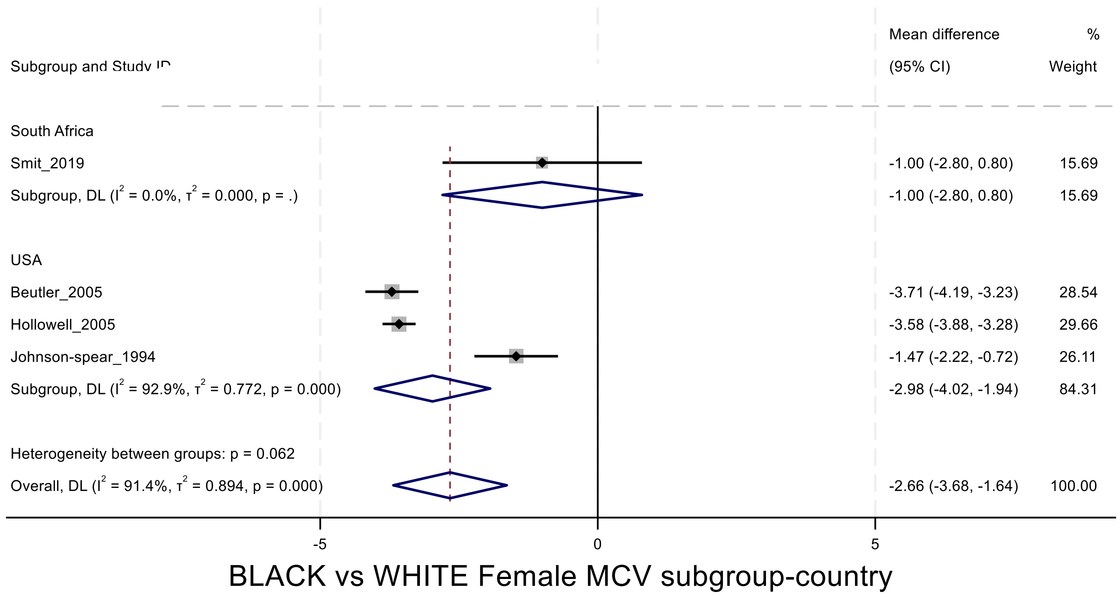

MCV Black vs White female (subgroup by study quality)

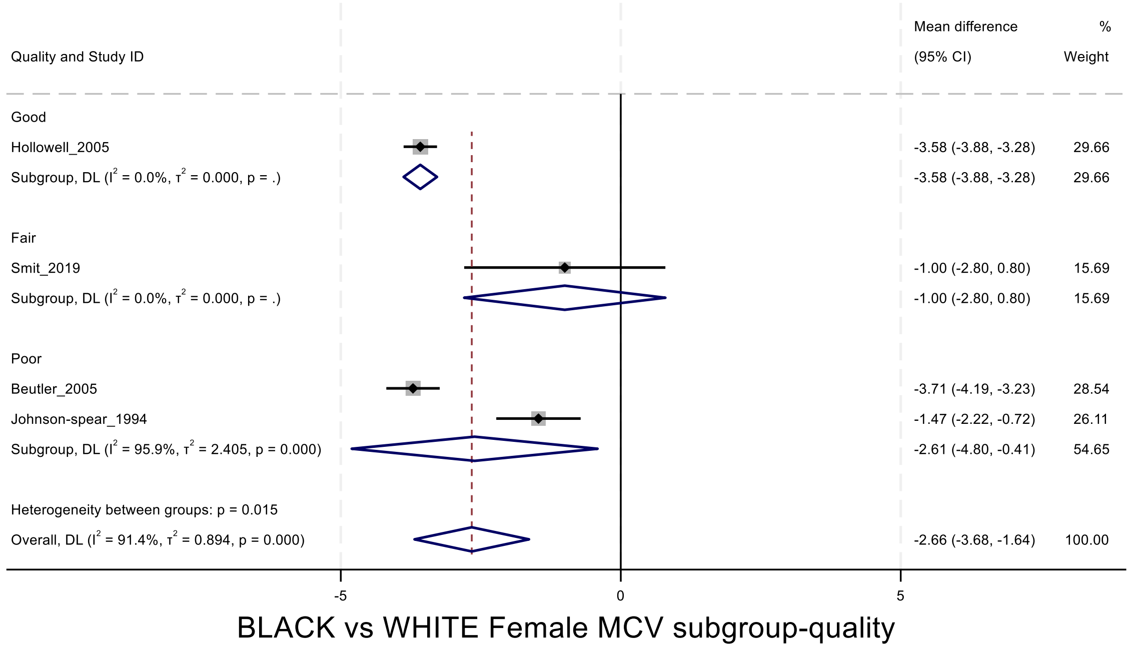

MCV Black vs White female (Fixed-effects model and random-effects model)

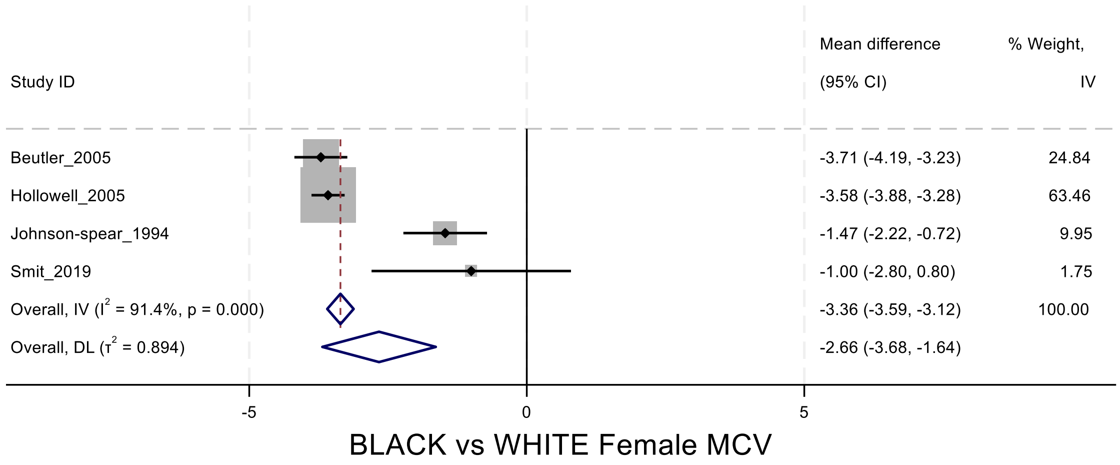

MCV Black vs White male (subgroup by country of study)

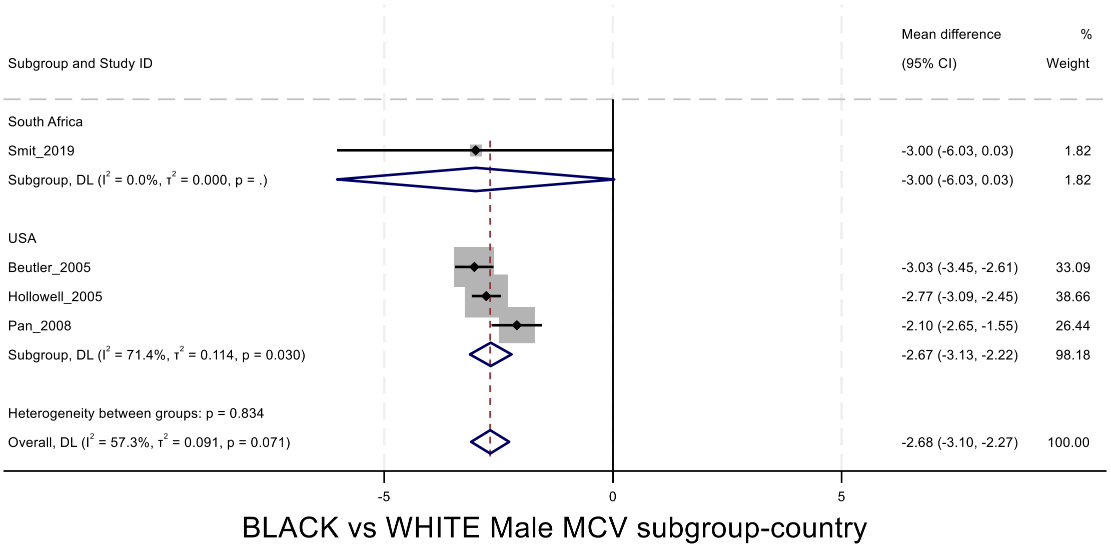

MCV Black vs White male (subgroup by study quality)

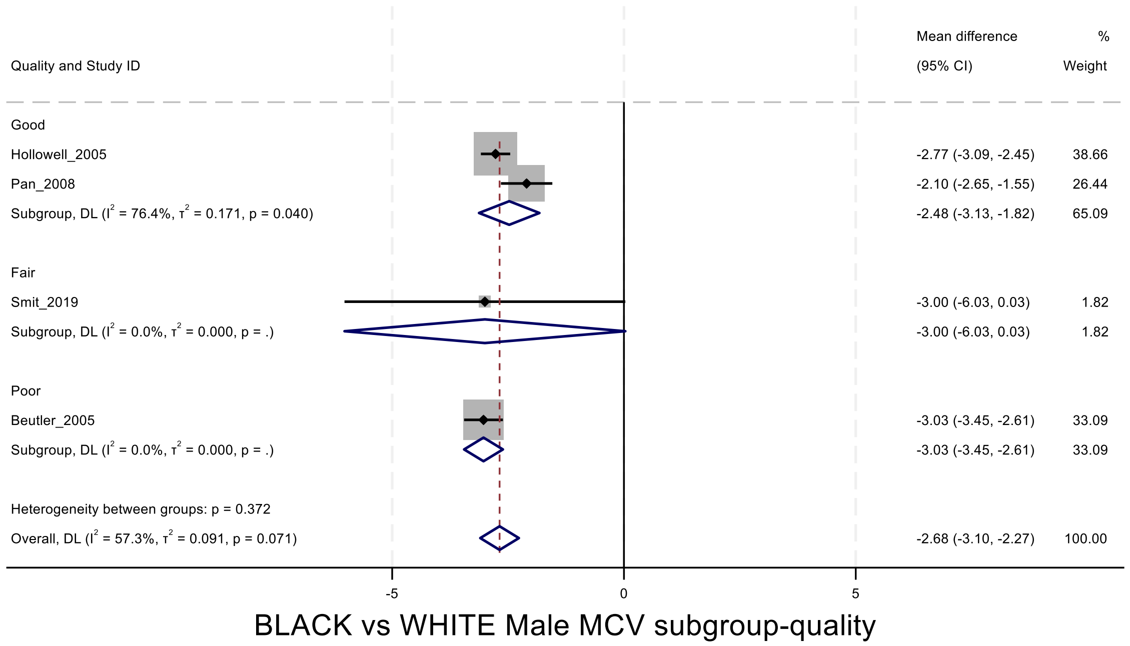

MCV Black vs White male (Fixed-effects model and random-effects model)

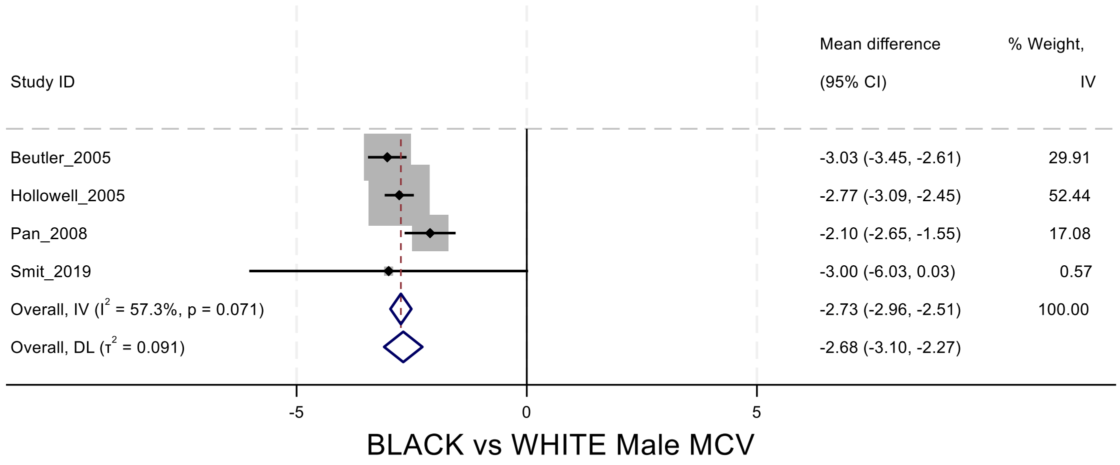

Platelet (PLT) Black vs White female (Fixed-effects model and random-effects model)

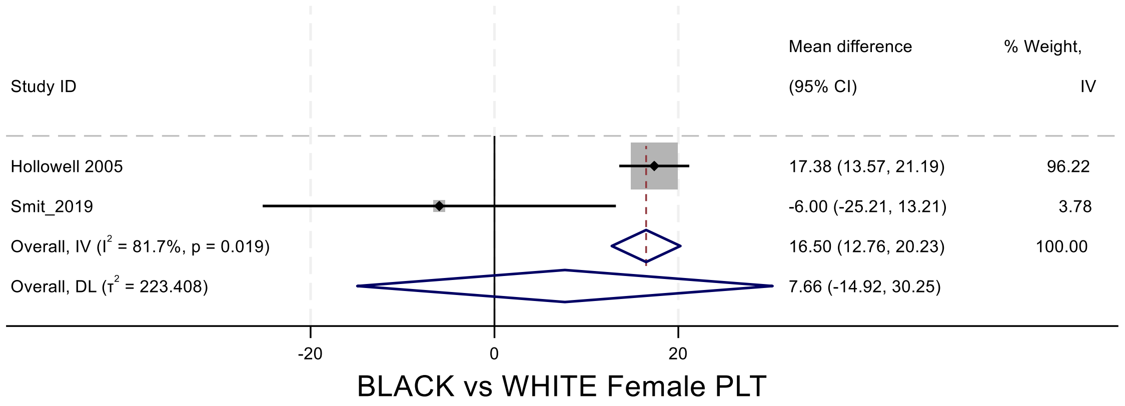

Platelet (PLT) Black vs White male (subgroup-country of study)

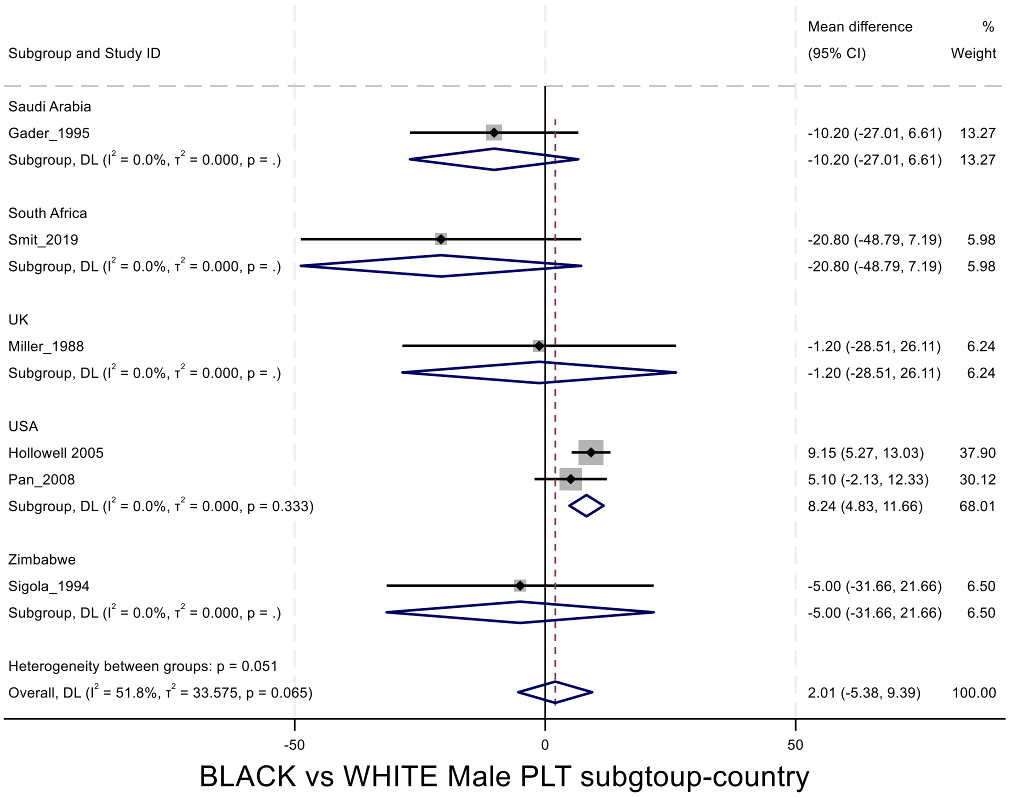

Platelet (PLT) Black vs White male (subgroup-study quality)

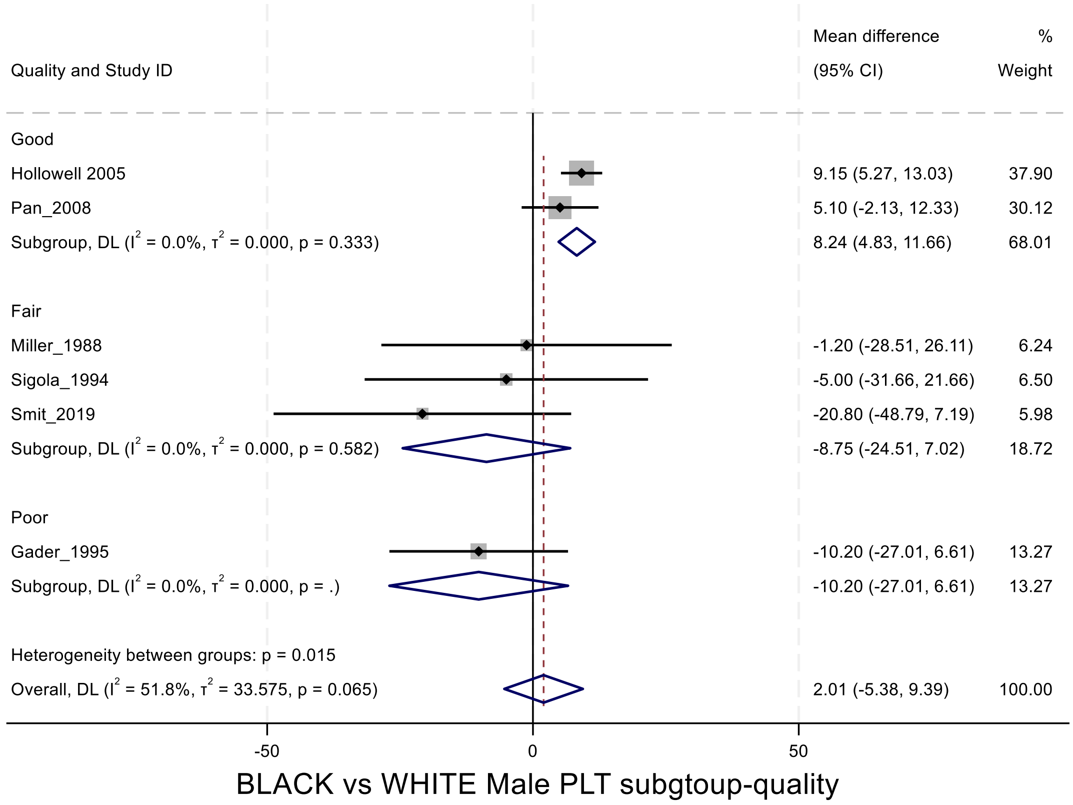

Platelet (PLT) Black vs White male (Fixed-effects model and random-effects model)

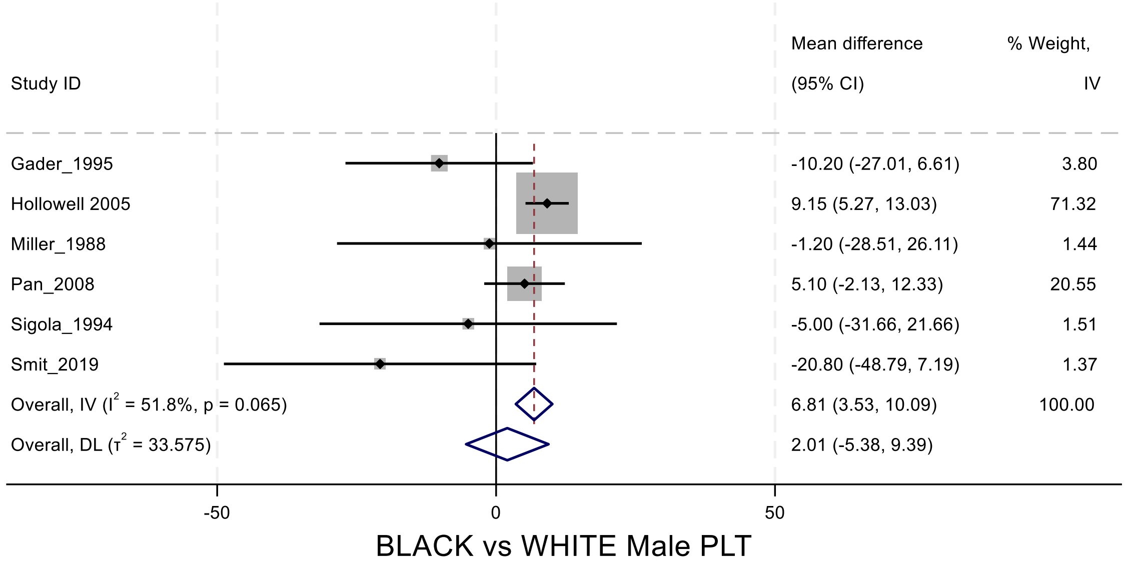

Platelet (PLT) Asian vs White male (Fixed-effects model and random-effects model)

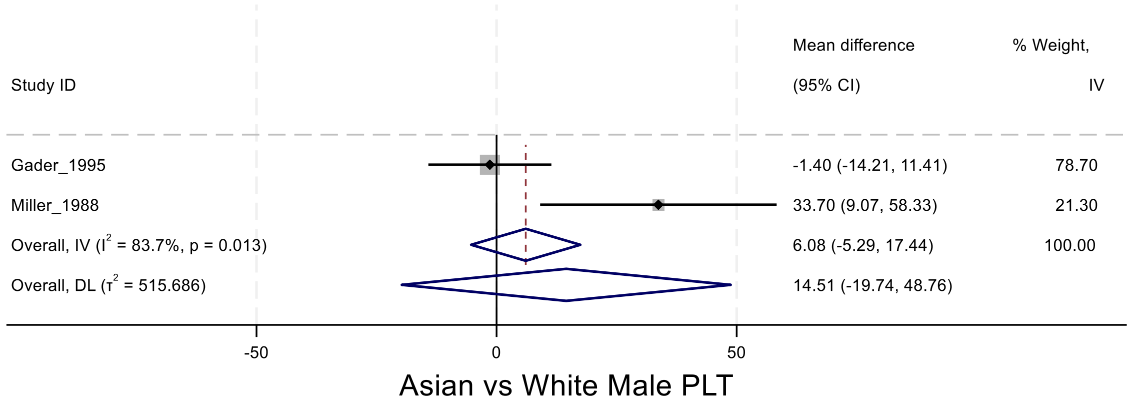

Platelet (PLT) Black vs Asian male (Fixed-effects model and random-effects model)

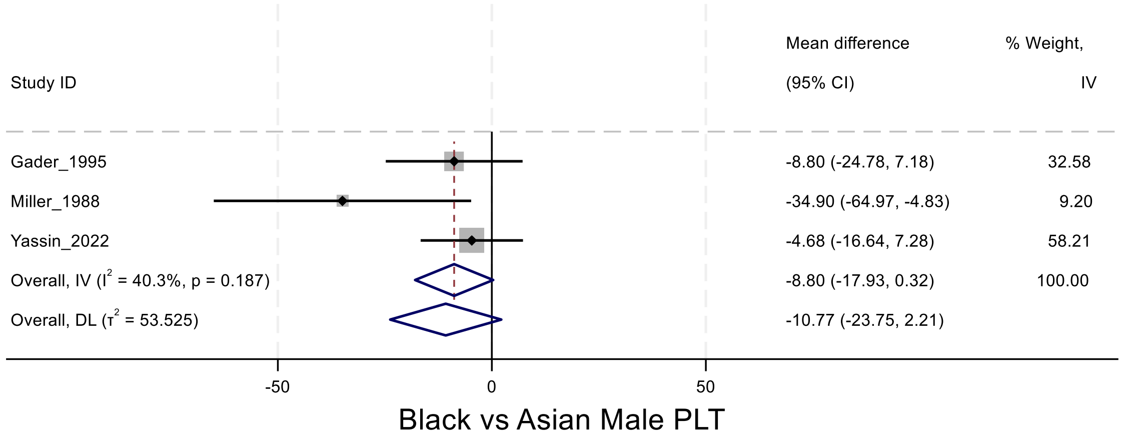

CRP Black vs White female (Fixed-effects model and random-effects model)

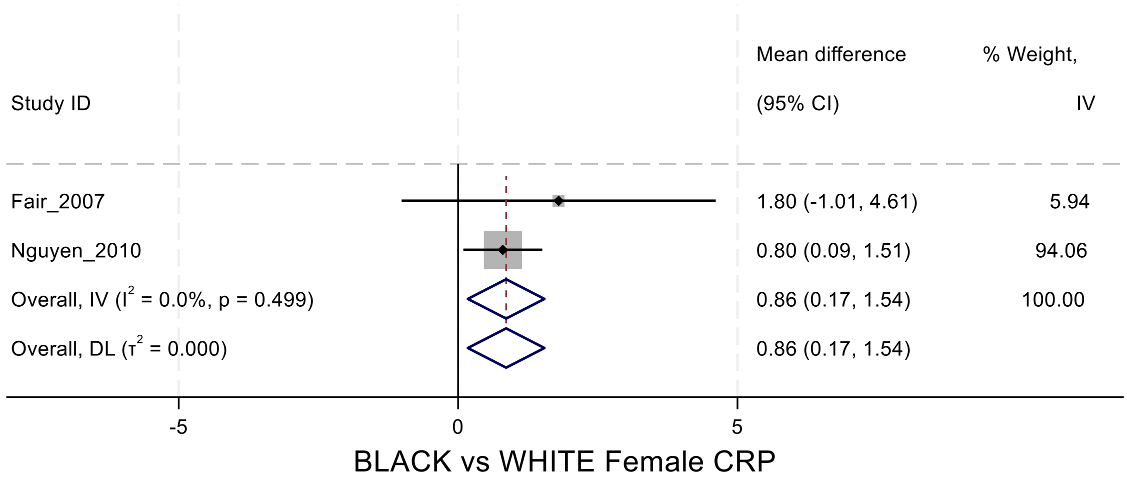

CRP Black vs White male (Fixed-effects model and random-effects model)

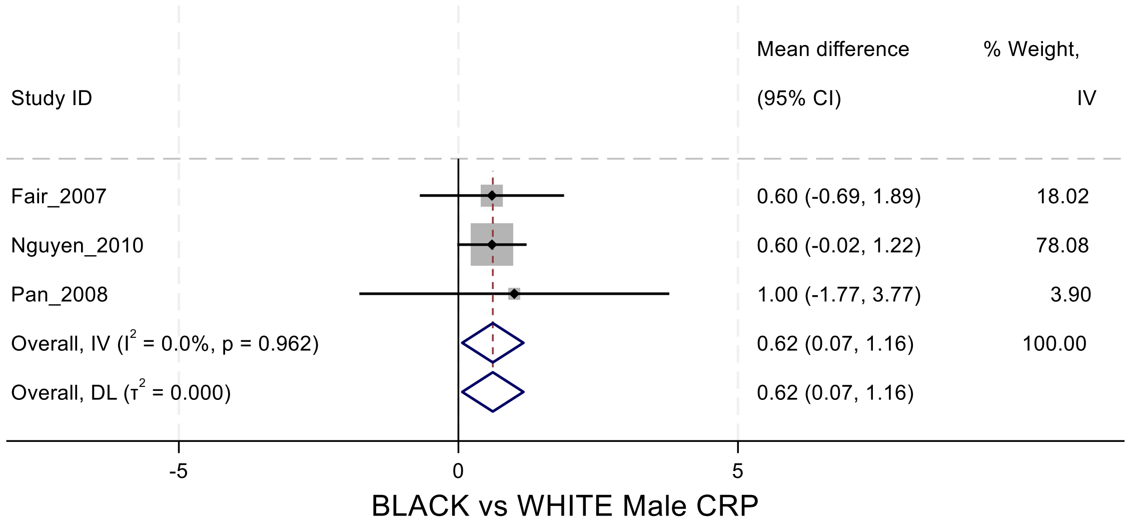

CRP Asian vs White female (Fixed-effects model and random-effects model)

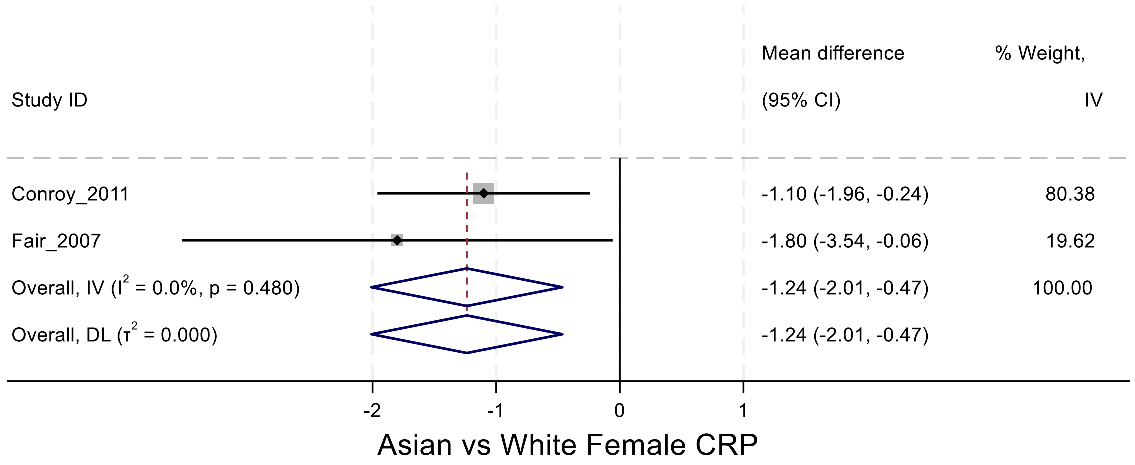

Calcium Black vs White female (Fixed-effects model and random-effects model)

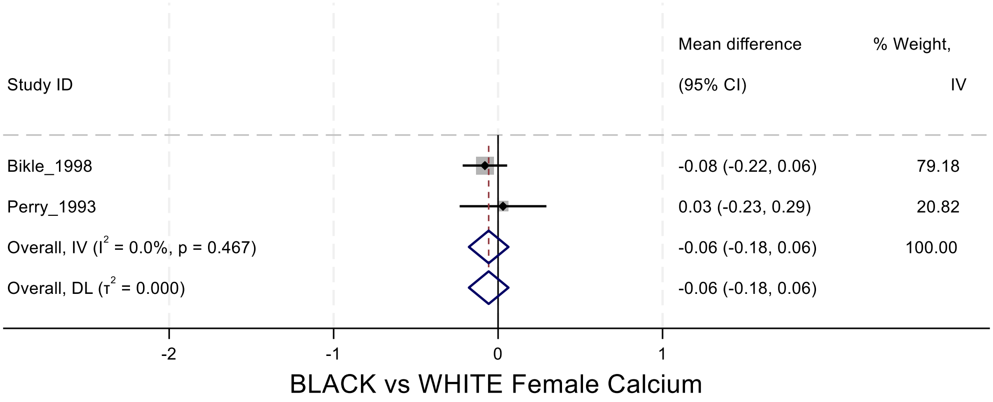

Calcium Black vs White male (Fixed-effects model and random-effects model)

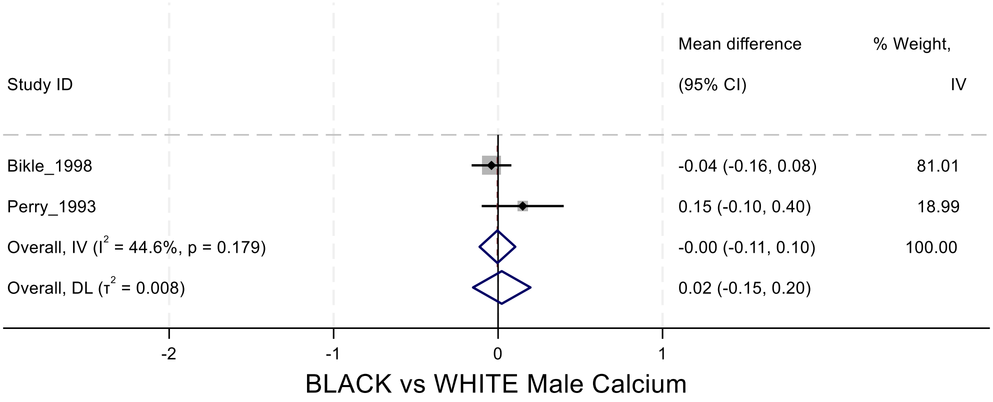

Albumin (ALB) Black vs White male (Fixed-effects model and random-effects model)

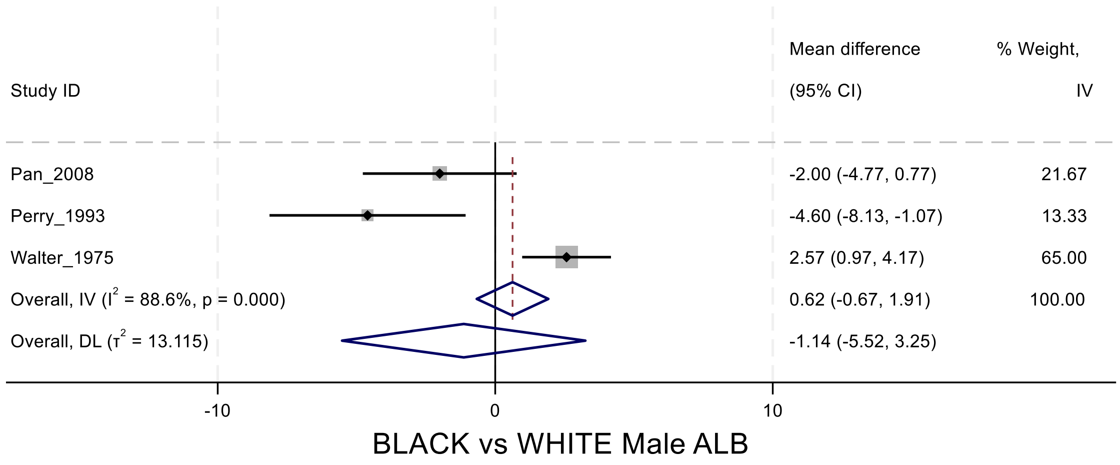

References

1. Beutler E, West C. Hematologic differences between African-Americans and whites: the roles of iron deficiency and alpha-thalassemia on hemoglobin levels and mean corpuscular volume. *Blood*. Jul 15 2005;106(2):740-5.
2. Buckle GC, Webb GL. Computer simulation as a method for evaluating the copper sulfate test for hemoglobin in diverse populations. *Transfusion*. May-Jun 1978;18(3):323-8.
3. Cheng CK, Chan J, Cembrowski GS, van Assendelft OW. Complete blood count reference interval diagrams derived from NHANES III: stratification by age, sex, and race. *Laboratory Hematology*. 2004;10(1):42-53.
4. Godsland IF, Seed M, Simpson R, Broom G, Wynn V. Comparison of haematological indices between women of four ethnic groups and the effect of oral contraceptives. *Journal of Clinical Pathology*. Feb 1983;36(2):184-91.
5. Hollowell JG, van Assendelft OW, Gunter EW, Lewis BG, Najjar M, Pfeiffer C. Hematological and iron-related analytes--reference data for persons aged 1 year and over: United States, 1988-94. *Vital and health statistics*. Mar 2005;Series 11, Data from the national health survey.(247):1-156.
6. Horn PS, Pesce AJ. Effect of ethnicity on reference intervals. *Clinical Chemistry*. Oct 2002;48(10):1802-4.
7. Jackson RT. Hemoglobin comparisons between African American and European American males with hemoglobin values in the normal range. *American Journal of Human Biology*. 1992;4(3):313-318. doi:https://dx.doi.org/10.1002/ajhb.1310040306
8. Jim RT. Red blood count, hemoglobin and red cell indices in healthy adult orientals in Hawaii. *Hawaii Medical Journal*. May 1987;46(5):158, 160, 163-6.
9. Johnson-Spear MA, Yip R. Hemoglobin difference between black and white women with comparable iron status: justification for race-specific anemia criteria. *American Journal of Clinical Nutrition*. Jul 1994;60(1):117-21.
10. Kerr GR, Lee ES, Lam MK, et al. Relationship between dietary and biochemical measures of nutritional status in HANES I data. *American Journal of Clinical Nutrition*. Feb 1982;35(2):294-307.
11. Kozlitina J, Garcia CK. Red blood cell size is inversely associated with leukocyte telomere length in a large multi-ethnic population. *PLoS ONE [Electronic Resource]*. 2012;7(12):e51046. doi:https://dx.doi.org/10.1371/journal.pone.0051046
12. Lawrie D, Coetzee LM, Becker P, Mahlangu J, Stevens W, Glencross DK. Local reference ranges for full blood count and CD4 lymphocyte count testing. *South African Medical Journal Suid-Afrikaanse Tydskrif Vir Geneeskunde*. Apr 2009;99(4):243-8.
13. Le CH. The Prevalence of Anemia and Moderate-Severe Anemia in the US Population (NHANES 2003-2012). *PLoS ONE [Electronic Resource]*. 2016;11(11):e0166635. doi:https://dx.doi.org/10.1371/journal.pone.0166635
14. Mast AE, Steele WR, Johnson B, et al. Population-based screening for anemia using first-time blood donors. *American Journal of Hematology*. 2012;doi:http://dx.doi.org/10.1002/ajh.23171
15. Miller GJ, Kotecha S, Wilkinson WH, et al. Dietary and other characteristics relevant for coronary heart disease in men of Indian, West Indian and European descent in London. *Atherosclerosis*. 1988;70(1-2):63-72.
16. Pan Y, Jackson RT. Insights into the ethnic differences in serum ferritin between black and white US adult men. *American Journal of Human Biology*. Jul-Aug 2008;20(4):406-16. doi:https://dx.doi.org/10.1002/ajhb.20745
17. Smit FC, Davison GM, Hoffmann M, Erasmus RT, Davids S, Matsha TE. Full blood count and white cell differential count reference ranges obtained from a healthy urban South African population residing in the Western Cape of South Africa. *International Journal of Laboratory Hematology*. Oct 2019;41(5):635-641. doi:https://dx.doi.org/10.1111/ijlh.13076
18. Thomson CA, Stanaway JD, Neuhouser ML, et al. Nutrient Intake and Anemia Risk in the Women's Health Initiative Observational Study. *Journal of the American Dietetic Association*. April 2011;111(4):532-541. doi:http://dx.doi.org/10.1016/j.jada.2011.01.017
19. Yassin MA, Soliman AT, Nashwan AJ, et al. Hematological indices reference intervals for a healthy Arab population in Qatar: Effect of age, gender, and geographic location. *Medicine*. 2022 2022;101(24):e29271. doi:doi:https://dx.doi.org/10.1097/MD.0000000000029271

20. Johnson AM, Petersen PH, Whicher JT, Carlstrom A, MacLennan S. Reference intervals for serum proteins: Similarities and differences between adult Caucasian and Asian Indian males in Yorkshire, UK. *Clinical Chemistry and Laboratory Medicine*. 2004;42(7):792-799. doi:http://dx.doi.org/10.1515/CCLM.2004.132
21. Bain BJ, Seed M. Platelet count and platelet size in healthy Africans and West Indians. *Clinical & Laboratory Haematology*. 1986;8(1):43-8.
22. Bain BJ. Ethnic and sex differences in the total and differential white cell count and platelet count. *Journal of Clinical Pathology*. Aug 1996;49(8):664-6.
23. Gader A, Bahakim H, Awadalla S, Malaika S. Ethnic variations in the haemostatic system: comparison between Arabs, Westerners (Europeans and Americans), Asians and Africans. *Blood Coagulation & Fibrinolysis*. Sep 1995;6(6):537-42.
24. Segal JB, Moliterno AR. Platelet counts differ by sex, ethnicity, and age in the United States. *Annals of Epidemiology*. Feb 2006;16(2):123-30.
25. Sigola LB, Mufunda J, Adewuyi JO, Keogh E. Factor VII, antithrombin III, leukocyte and platelet counts in a sample of black and white Zimbabwean blood donors. *Thrombosis Research*. Jul 01 1994;75(1):73-9.
26. Akinyemiju T, Moore JX, Pisu M, et al. Association of baseline inflammatory biomarkers with cancer mortality in the REGARDS cohort. *Oncotarget*. 01 Aug 2019;10(47):4857-4867.
27. Albert MA, Glynn RJ, Buring J, Ridker PM. C-reactive protein levels among women of various ethnic groups living in the United States (from the Women's Health Study). *American Journal of Cardiology*. May 15 2004;93(10):1238-42.
28. Anand SS, Razak F, Yi Q, et al. C-reactive protein as a screening test for cardiovascular risk in a multiethnic population. *Arteriosclerosis, Thrombosis & Vascular Biology*. Aug 2004;24(8):1509-15.
29. Beasley LE, Koster A, Newman AB, et al. Inflammation and race and gender differences in computerized tomography-measured adipose depots. *Obesity*. May 2009;17(5):1062-1069. doi:http://dx.doi.org/10.1038/oby.2008.627
30. Chandalia M, Cabo-Chan AV, Jr., Devaraj S, Jialal I, Grundy SM, Abate N. Elevated plasma high-sensitivity C-reactive protein concentrations in Asian Indians living in the United States. *Journal of Clinical Endocrinology & Metabolism*. Aug 2003;88(8):3773-6.
31. Conroy SM, Chai W, Lim U, Franke AA, Cooney RV, Maskarinec G. Leptin, adiponectin, and obesity among Caucasian and Asian women. *Mediators of Inflammation*. 2011;2011:253580. doi:https://dx.doi.org/10.1155/2011/253580
32. Fair JM, Kiazand A, Varady A, et al. Ethnic differences in coronary artery calcium in a healthy cohort aged 60 to 69 years. *American Journal of Cardiology*. Sep 15 2007;100(6):981-5.
33. Ford ES. Does exercise reduce inflammation? Physical activity and C-reactive protein among U.S. adults. *Epidemiology*. Sep 2002;13(5):561-8.
34. Ford ES, Giles WH, Mokdad AH, Myers GL. Distribution and Correlates of C-Reactive Protein Concentrations among Adult US Women. *Clinical Chemistry*. March 2004;50(3):574-581. doi:http://dx.doi.org/10.1373/clinchem.2003.027359
35. Hanley AJ, Bowden D, Wagenknecht LE, et al. Associations of adiponectin with body fat distribution and insulin sensitivity in nondiabetic Hispanics and African-Americans. *Journal of Clinical Endocrinology & Metabolism*. Jul 2007;92(7):2665-71.
36. Khera A, McGuire DK, Murphy SA, et al. Race and gender differences in C-reactive protein levels. *Journal of the American College of Cardiology*. Aug 02 2005;46(3):464-9.
37. Lin SX, Pi-Sunyer EX. Prevalence of the metabolic syndrome among US middle-aged and older adults with and without diabetes--a preliminary analysis of the NHANES 1999-2002 data. *Ethnicity & Disease*. 2007;17(1):35-9.
38. Matthews KA, Sowers MF, Derby CA, et al. Ethnic differences in cardiovascular risk factor burden among middle-aged women: Study of Women's Health Across the Nation (SWAN). *American Heart Journal*. Jun 2005;149(6):1066-73.
39. Nguyen QM, Srinivasan SR, Xu JH, Chen W, Berenson GS. Distribution and cardiovascular risk correlates of plasma soluble intercellular adhesion molecule-1 levels in asymptomatic young adults from a biracial community: the Bogalusa Heart Study. *Annals of Epidemiology*. Jan 2010;20(1):53-9. doi:https://dx.doi.org/10.1016/j.annepidem.2009.10.001

40. Wener MH, Daum PR, McQuillan GM. The influence of age, sex, and race on the upper reference limit of serum C-reactive protein concentration. *Journal of Rheumatology*. Oct 2000;27(10):2351-9.
41. Birk R, Heifetz EM. Variations in biochemical values for common laboratory tests: a comparison among multi-ethnic Israeli women cohort. *Irish Journal of Medical Science*. Feb 2019;188(1):249-258. doi:https://dx.doi.org/10.1007/s11845-018-1825-y
42. Manolio TA, Burke GL, Savage PJ, et al. Sex- and race-related differences in liver-associated serum chemistry tests in young adults in the CARDIA study. *Clinical Chemistry*. Sep 1992;38(9):1853-9.
43. Perry HM, 3rd, Miller DK, Morley JE, et al. A preliminary report of vitamin D and calcium metabolism in older African Americans. *Journal of the American Geriatrics Society*. Jun 1993;41(6):612-6.
44. Walter H, Schoebel B. Climate associated variations in the human serum albumin level. *Humangenetik*. 1975;30(4):331-335.
45. Bikle DD, Ettinger B, Sidney S, Tekawa IS, Tolan K. Differences in calcium metabolism between black and white men and women. *Mineral & Electrolyte Metabolism*. May-Jun 1999;25(3):178-84.
46. Brickman AS, Nyby MD, Griffiths RF, von Hungen K, Tuck ML. Racial differences in platelet cytosolic calcium and calcitropic hormones in normotensive subjects. *American Journal of Hypertension*. Jan 1993;6(1):46-51.
47. Hamson C, Goh L, Sheldon P, Samanta A. Comparative study of bone mineral density, calcium, and vitamin D status in the Gujarati and white populations of Leicester. *Postgraduate Medical Journal*. May 2003;79(931):279-83.
